# Measuring similarities between gene expression profiles through new data transformations

**DOI:** 10.1186/1471-2105-8-29

**Published:** 2007-01-27

**Authors:** Kyungpil Kim, Shibo Zhang, Keni Jiang, Li Cai, In-Beum Lee, Lewis J Feldman, Haiyan Huang

**Affiliations:** 1 Department of Statistics, University of California, Berkeley, USA; 2 Department of Chemical Engineering, Pohang University of Science and Technology (POSTECH), Korea; 3 Department of Plant and Microbial Biology, University of California, Berkeley, USA; 4 Department of Biomedical Engineering, Rutgers University, USA

## Abstract

**Background:**

Clustering methods are widely used on gene expression data to categorize genes with similar expression profiles. Finding an appropriate (dis)similarity measure is critical to the analysis. In our study, we developed a new measure for clustering the genes when the key factor is the shape of the profile, and when the expression magnitude should also be accounted for in determining the gene relationship. This is achieved by modeling the shape and magnitude parameters separately in a gene expression profile, and then using the estimated shape and magnitude parameters to define a measure in a new feature space.

**Results:**

We explored several different transformation schemes to construct the feature spaces that include a space whose features are determined by the mutual differences of the original expression components, a space derived from a parametric covariance matrix, and the principal component space in traditional PCA analysis. The former two are the newly proposed and the latter is explored for comparison purposes. The new measures we defined in these feature spaces were employed in a *K*-means clustering procedure to perform analyses. Applying these algorithms to a simulation dataset, a developing mouse retina SAGE dataset, a small yeast sporulation cDNA dataset, and a maize root affymetrix microarray dataset, we found from the results that the algorithm associated with the first feature space, named *TransChisq*, showed clear advantages over other methods.

**Conclusion:**

The proposed *TransChisq *is very promising in capturing meaningful gene expression clusters. This study also demonstrates the importance of data transformations in defining an efficient distance measure. Our method should provide new insights in analyzing gene expression data. The clustering algorithms are available upon request.

## Background

With the explosion of various 'omic' data, a general question facing the biologists and statisticians is how to summarize and organize the observed data into meaningful structures. Clustering is one of the methods that have been widely explored for this purpose [1-3]. In particular, clustering is being generally applied to gene expression data to group genes with similar expression profiles into discrete functional clusters. Many clustering methods are available, including hierarchical clustering [4], *K*-means clustering [5], self-organizing maps [6], and various model-based methods [7-9].

Recent research in clustering analysis has been focused largely on two areas: estimating the number of clusters in data [10-12] and the optimization of the clustering algorithms [13,14]. In this paper we studied a different yet fundamental issue in clustering analysis: to define an appropriate measure of similarity for gene expression patterns.

The most commonly used distances or similarity measures for analyzing gene expression data are the *Pearson correlation coefficient *and *Euclidean distance*, which however, in some situations, could be unsuitable to explore the true gene relationship. The *Pearson correlation coefficient *is overly sensitive to the shape of an expression curve, and the *Euclidean distance *mainly considers the magnitude of the changes of the gene expression. For other model-based methods [7-9,15], their successes would highly rely on how well the assumed probability model fits the data and the clustering purpose.

In recent literature, several advanced measures with emphasis on the expression profile shape have been developed in different contexts [16-18]. In particular, based on the *Spearman Rank Correlation*, *CLARITY *was defined for detecting the local similarity or time-shifted patterns in expression profiles [18]. However, the rank-based methods could mistakenly interpret a pattern since the use of rank causes information loss. As an example, we consider a profile ***Y ***= (104, 95, 88, 92, 88) with all components generated from the same Poisson distribution of mean 100. Clearly, the differences among the components in ***Y ***are due to the distribution variance and ranking in this case is meaningless. Briefly, *Spearman Rank Correlation *cannot distinguish the real differences from random errors in some situations and thus may provide a wrong estimate of the pattern.

By separately modeling the shape and the magnitude parameters in a gene expression profile, we developed a new measure for clustering the genes when the profile shape is a key factor, and when the expression magnitude should also be accounted for in determining the gene relationship. The approach is to use the estimated shape and magnitude parameters to define a Chi-square-statistic based distance measure in a new feature space. An appropriate feature space helps summarize the data more effectively, hence improving the identification of gene relationships. We explored different transformation schemes to construct the feature spaces, which include a space with features determined by the mutual differences of the original expression components, a space derived from a parametric covariance matrix, and the principal component space in PCA analysis [19]. The former two are the newly proposed and the latter is explored for comparison purposes.

The new measures associated with different feature spaces were employed in a *K*-means clustering procedure to perform clustering analyses. We designated the algorithm using the measure from the first transformed space as *TransChisq*, and the one associated with the principal component space as *PCAChisq*. The space derived from a parametric covariance matrix is not included in comparison for computational reasons (see Methods). For evaluation purposes we also implemented a set of widely used measures into the *K*-means clustering procedure, including Pearson correlation coefficient (*PearsonC*), Euclidian distance (*Eucli*), Spearman Rank Correlation (*SRC*), and a chi-square based measure for Poisson distributed data (*PoissonC*) [20]. All the measures were applied to a simulation dataset, a developing mouse retina SAGE dataset of 153 tags [21], a small yeast sporulation cDNA dataset [22], and a maize root affymetrix microarray dataset [23]. The results showed that *TransChisq *outperforms other methods. Using the gap statistic [24,25], *TransChisq *was also found to be advantageous in estimating the number of clusters. The underlying probability model of our method was adopted from the model of *PoissonC*, a method for analyzing the expression patterns in Serial Analysis of Gene Expression (SAGE) data [20]. The MATLAB source codes for all these algorithms are available upon request.

## Results

First, we will illustrate the property of the proposed new transformations by applying them to a maize gene expression dataset. Then we will present the applications of *TransChisq*, *PCAChisq *and other methods to a simulation dataset, a yeast sporulation microarray dataset, and a mouse retinal SAGE dataset.

### Experimental maize gene expression data

The maize dataset, consisting of nine Affymetrix microarrays, was generated to investigate the gene transcription activity in three maize root tissues with three replicates for each tissue: the proximal meristem (PM), the quiescent center (QC) and the root cap (RC) [23]. 2092 significantly differentially expressed genes have been identified and categorized into 6 classes of expression patterns [23]. Here we used these genes to illustrate the property of the proposed transformations with comparison to the traditional PCA.

**Figure 1:**
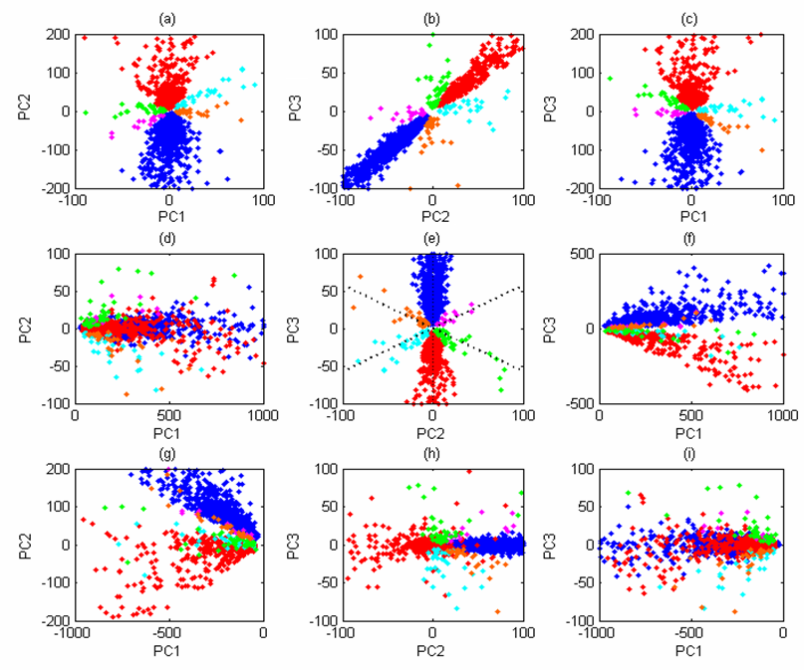
**Plots of 2092 maize genes on to the three different feature spaces**. From top to bottom, the genes are plotted on to the subspaces of dimension 2 of the new spaces. Figures 1(a-c) correspond to the space used in *TransChisq*, Figures 1(d-f) correspond to the space determined by the parametric covariance matrix and Figures 1(g-i) correspond to the principal component space associated with the *PCAChisq*. PC1, PC2 and PC3 specify the subspaces. Blue/red dots represent RC up-/down-regulated genes, cyanide/pink dots represent PM up-/down-regulated genes, green/orange dots represent QC up-/down-regulated genes. The dotted lines in (e) are the centers of the six class separating regions determined by the second and third component from the parametric covariance matrix.

Firstly, we applied the transformation employed in *TransChisq *to the data. Figures 1(a)–(c) plot the expression profiles of the genes in this new space. The blue and red genes are from the two dominant classes (RC up- or down-regulated genes account for 94% of all genes) and the other four colors (orange, green, pink, light blue) correspond to the other four small classes (up- or down-regulated genes in QC or PM account for 6% of all genes). The three plots show that the six classes can be recognized explicitly in any of the three subspaces of dimension 2.

We then applied the transformation suggested by a parametric covariance matrix to the same data (see Methods). Figures 1(d)–(f) plot the expression profiles of the genes in this new space. We can see that the second and the third component separate all six classes in Figure 1(e) correctly. The description of the six class separating regions, whose centers are the dotted lines in Figure 1(e), is in Table 1 (e.g., the genes around the line PC2 = 3
 MathType@MTEF@5@5@+=feaafiart1ev1aaatCvAUfKttLearuWrP9MDH5MBPbIqV92AaeXatLxBI9gBaebbnrfifHhDYfgasaacH8akY=wiFfYdH8Gipec8Eeeu0xXdbba9frFj0=OqFfea0dXdd9vqai=hGuQ8kuc9pgc9s8qqaq=dirpe0xb9q8qiLsFr0=vr0=vr0dc8meaabaqaciaacaGaaeqabaqabeGadaaakeaadaGcaaqaaiabiodaZaWcbeaaaaa@2DBB@·PC3 < 0 are expected to be PM up-regulated). A convenient common property of this transformation, and the one in *TransChisq*, is that the information carried by each component is explicit, and hence the region in the new space corresponding to each class can be clearly determined.

**Table 1:** The six expression patterns and their separating regions described by PC2 and PC3

Class index	Expression patterns	Center of separating regions described by PC2 and PC3
1	PM > (QC ≈ RC)	PC2 = 3 MathType@MTEF@5@5@+=feaafiart1ev1aaatCvAUfKttLearuWrP9MDH5MBPbIqV92AaeXatLxBI9gBaebbnrfifHhDYfgasaacH8akY=wiFfYdH8Gipec8Eeeu0xXdbba9frFj0=OqFfea0dXdd9vqai=hGuQ8kuc9pgc9s8qqaq=dirpe0xb9q8qiLsFr0=vr0=vr0dc8meaabaqaciaacaGaaeqabaqabeGadaaakeaadaGcaaqaaiabiodaZaWcbeaaaaa@2DBB@·PC3 < 0
2	PM < (QC ≈ RC)	PC2 = 3 MathType@MTEF@5@5@+=feaafiart1ev1aaatCvAUfKttLearuWrP9MDH5MBPbIqV92AaeXatLxBI9gBaebbnrfifHhDYfgasaacH8akY=wiFfYdH8Gipec8Eeeu0xXdbba9frFj0=OqFfea0dXdd9vqai=hGuQ8kuc9pgc9s8qqaq=dirpe0xb9q8qiLsFr0=vr0=vr0dc8meaabaqaciaacaGaaeqabaqabeGadaaakeaadaGcaaqaaiabiodaZaWcbeaaaaa@2DBB@·PC3 > 0
3	QC > (PM ≈ RC)	PC2 = -3 MathType@MTEF@5@5@+=feaafiart1ev1aaatCvAUfKttLearuWrP9MDH5MBPbIqV92AaeXatLxBI9gBaebbnrfifHhDYfgasaacH8akY=wiFfYdH8Gipec8Eeeu0xXdbba9frFj0=OqFfea0dXdd9vqai=hGuQ8kuc9pgc9s8qqaq=dirpe0xb9q8qiLsFr0=vr0=vr0dc8meaabaqaciaacaGaaeqabaqabeGadaaakeaadaGcaaqaaiabiodaZaWcbeaaaaa@2DBB@·PC3 > 0
4	QC < (PM ≈ RC)	PC2 = -3 MathType@MTEF@5@5@+=feaafiart1ev1aaatCvAUfKttLearuWrP9MDH5MBPbIqV92AaeXatLxBI9gBaebbnrfifHhDYfgasaacH8akY=wiFfYdH8Gipec8Eeeu0xXdbba9frFj0=OqFfea0dXdd9vqai=hGuQ8kuc9pgc9s8qqaq=dirpe0xb9q8qiLsFr0=vr0=vr0dc8meaabaqaciaacaGaaeqabaqabeGadaaakeaadaGcaaqaaiabiodaZaWcbeaaaaa@2DBB@·PC3 < 0
5	RC > (PM ≈ QC)	PC2 = 0; PC3 > 0
6	RC < (PM ≈ QC)	PC2 = 0; PC3 < 0

For comparison, we performed a traditional PCA analysis to the same data. Figures 1(g)–(i) plot the expression profiles of the genes in the principal component space. We can see that the direct application of the PCA can separate the two dominating expression patterns. But it fails to recognize the other patterns, even when exhausting all principal components. The poor performance of PCA could be attributed to the use of empirical sample covariance matrix in determining the principal components. In the maize dataset, about 94% genes are RC up- or down-regulated genes, which cause most of the variance. The principal components, determined by this sample covariance matrix thus largely capture the two dominating clusters, yet miss the meaningful class information for the other four small groups.

This example demonstrates the advantage of the proposed new data transformations over the traditional PCA in keeping class information intact.

### Simulation study

We applied *TransChisq *to a simulation dataset to evaluate its performance. For comparison purposes, other modified *K*-means algorithms, i.e. *PCAChisq*, *PoissonC*, *PearsonC*, and *Eucli *were also applied to the same dataset.

The simulation dataset consists of 46 vectors of dimension 5 and the components are independently generated from different Normal distributions. The mean (*μ*) and variance (*σ*^2^) of the Normal distributions are constrained by *σ*^2 ^= 3*μ *and described in Table 2. Based on the Normal distributions they are generated from, the 46 vectors are put into six groups, i.e., A, B, C, D, E, and F, whose sizes are 3, 6, 6, 9, 7, and 15 respectively. The motivation and guideline on choosing the various parameters related to this simulation datasets are presented in Additional file 1. Genes with a similar expression shape are considered to be in the same group. Although the expression magnitude in the dataset is not a critical factor for determining the gene clusters, its information is useful and should be taken into account when comparing the profile shapes.

**Table 2:** Five dimensional simulation dataset with Normal distributions (*σ*^2 ^= 3*μ*).

Group ID		Mean parameters of the Normal distributions (*μ*)				
Group A	a1 ~ a3	1	1	1	15	150
Group B	b1 ~ b6	15	1	1	1	150
Group C	c1 ~ c4	10	30	30	60	10
	c5 ~ c6	100	300	300	600	100
Group D	d1 ~ d7	200	70	70	10	10
	d8 ~ d9	2000	700	700	100	100
Group E	e1 ~ e5	210	120	10	10	10
	e6 ~ e7	2100	1200	100	100	100
Group F	f1 ~ f3	5	50	5	5	5
	f4 ~ f6	5	75	5	5	5
	F7 ~ f9	5	100	5	5	5
	f10 ~ f11	50	500	50	50	50
	f12 ~ f13	50	750	50	50	50
	f14 ~ f15	50	1000	50	50	50

**Figure 2:**
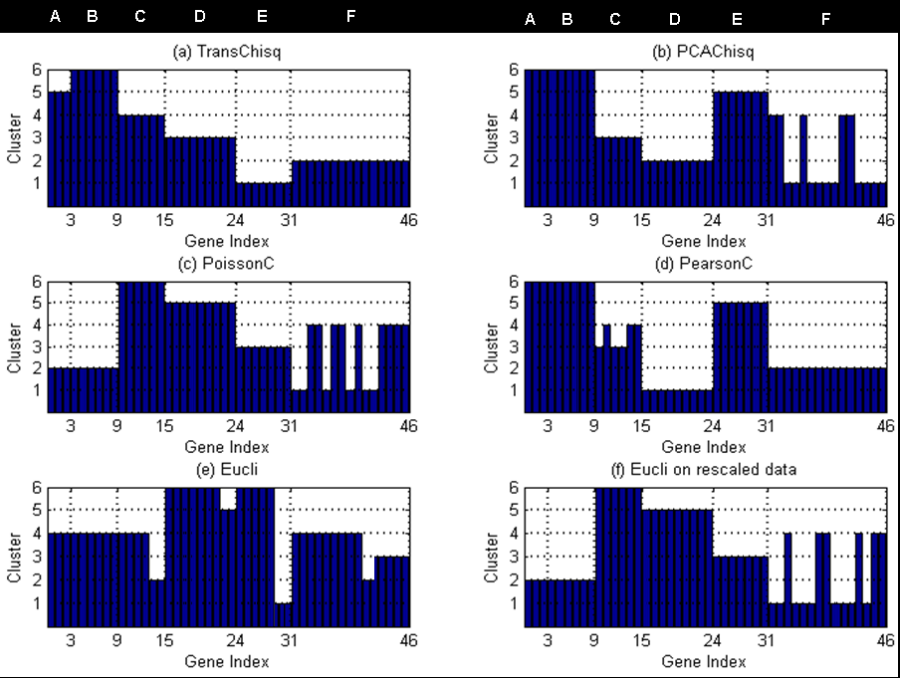
**Graphs of clustering results for the simulation data**. Horizontal axis represents the index of the 46 genes which belong to six groups (designated A, B, C, D, E and F, and marked at the top of the figure); vertical axis represents the index of the cluster that each gene has been assigned to by each algorithm.

The clustering results from different methods are shown in Figure 2. The horizontal axis represents the index of the 46 genes that belong to six groups (designated A, B, C, D, E and F, and marked at the top of the figure). The vertical axis represents the index of the cluster to which each gene has been assigned by a particular algorithm. Only *TransChisq *correctly categorized the genes into six groups. *PCAChisq*, *PoissonC*, and *PearsonC *mixed up group A and group B. *Eucli *clustered genes mainly by the magnitude of the gene expression values rather than the changes of the profile shapes. To reduce the effects from the magnitude, we further applied *Eucli *to the rescaled data. The rescaling was performed in a way so that the sum of the components within each vector was set the same. The clustering result of *Eucli *on the rescaled data (Figure 2(f)) is better, but not perfect.

We performed an additional 100 replications of the above simulation. *TransChisq*, *PCAChisq *and *PoissonC *correctly clustered 75, 37 and 43 of the 100 replicate simulation datasets, while *PearsonC*, *Eucli *and *Eucli *on rescaled data cannot generate correct clusters. We also tried *PCAChisq *on different combinations of principal components to optimize the clustering results. These different combinations, however, are not helpful to identify all the six groups.

This study evaluates the performance of *TransChisq *on the normally distributed data with Poisson-like property: variance increases with mean. The success of this application sheds a light on applying *TransChisq *to a microarray dataset in addition to the SAGE data.

### Experimental mouse retinal SAGE data

**Figure 3:**
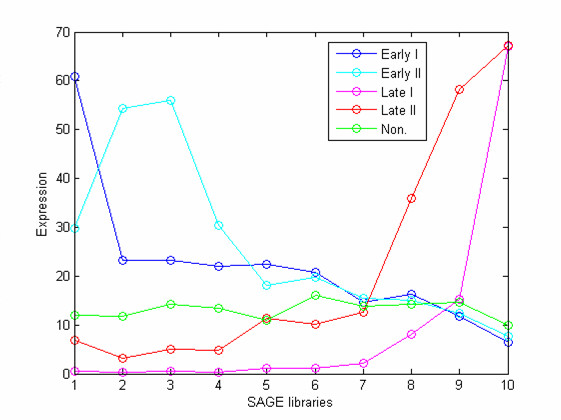
**Average expression profiles for the 153 SAGE tags**. These 153 tags fall into 5 clusters: 125 of these genes are developmental genes and can be further categorized into four groups (Early I, Early II, Late I and Late II) by their expressions at different developmental stages; the other 28 genes are not relevant to the mouse retina development.

For further validation we applied *TransChisq*, *PCAChisq*, *PoissonC*, *PearsonC*, *Eucli *and *SRC *(the *K*-means algorithm using Spearman Rank correlation as the similarity measure) to a set of mouse retinal SAGE libraries. The raw mouse retinal data consists of 10 SAGE libraries (38818 unique tags with tag counts ≥ 2) from developing retina taken at 2-day intervals. The samples range from embryonic, to postnatal, to adult [21]. Among the 38818 tags, 1467 tags that have counts greater than or equal to 20 in at least one of the 10 libraries were selected. The purpose of this selection is to exclude the genes with uniform low expression. To be more effective in comparing the clustering algorithms, a subset of 153 SAGE tags with known biological functions were selected. These 153 tags fall into 5 functional groups: 125 of these genes are developmental genes that can be further categorized into four classes by their activities at different developmental stages; the other 28 genes are not relevant to the mouse retina development (see Table 3). The average expression profile for each of the five clusters is shown in Figure 3.

**Table 3:** Functional categorization of the 153 mouse retinal tags (125 developmental genes; 28 non-developmental genes).

	Function Groups					
		
	Early I	Early II	Late I	Late II	Non-dev.	Total
Number of tags	32	34	32	27	28	153

*TransChisq*, *PCAChisq*, *PoissonC*, *PearsonC*, *Eucli *and *SRC *were applied to group these 153 SAGE tags into five clusters. Here we assumed that the number of the clusters, *K*, is known. A study to evaluate the performance of different measures in determining *K *when it is unknown can be found in a later section of this paper. The clustering results showed that *TransChisq *and *PCAChisq *outperform others (Table 4): 12, 12, 22, 26 and 38 of the 153 tags are incorrectly clustered by *TransChisq*, *PCAChisq*, *PoissonC*, *PearsonC *and *Eucli *on rescaled data respectively. For the results from *Eucli *on original data, as the correspondence between the predicted clusters and the true clusters is unclear, we cannot report the number of incorrectly clustered tags. We also evaluated the quality of the clustering results against an external criterion, the adjusted Rand Index [26]. The adjusted Rand Index assesses the degree of agreement between two partitions of the same set of objects. We compared the clustering results from each algorithm with the true categorizations, and calculated the adjusted Rand Index accordingly. The adjusted Rand Index varies between 1 (when the two partitions are identical) and 0 (when the partitions or the resulted clusters are random). A higher adjusted Rand Index represents the higher correspondence between the two partitions. From Table 4, we can see that the adjusted Rand Index results confirm that *TransChisq *and *PCAChisq *perform similarly and have clear advantages over other methods.

**Table 4:** Comparison of the algorithms on the 153 SAGE tags

Algorithm	Number of tags in incorrect clusters	% of tags in incorrect clusters	Adjusted Rand Index
*TransChisq*	12	7.8	0.822
*PCAChisq*	12	7.8	0.825
*PoissonC*	22	14.4	0.725
*PearsonC*	26	17.0	0.664
*Eucli*	NA	NA	0.003
*Eucli *on rescaled data	38	24.8	0.675
*SRC*	NA	NA	0.347

### Microarray yeast sporulation gene expression data

**Figure 4:**
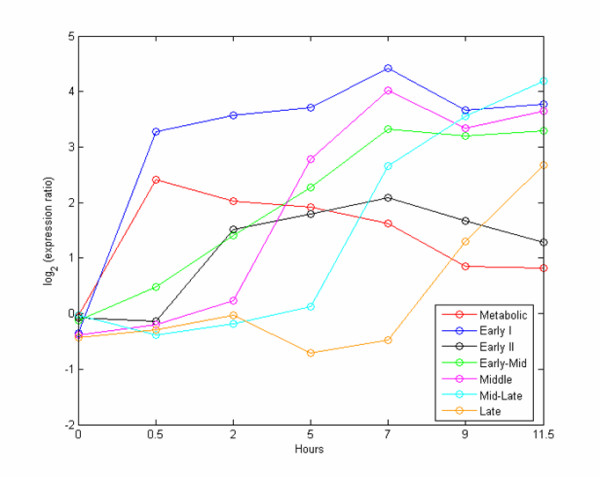
**Expression patterns of the 39 representative genes in the yeast sporulation data**. These 39 representative genes represent seven expression patterns in the yeast sporulation data. The figure shows the average expression profile for each pattern.

To further demonstrate how effective *TransChisq *is in clustering genes with characterized patterns in a microarray analysis, we applied *TransChisq *to a microarray yeast sporulation dataset [22]. Chu et al. measured gene expressions in the budding yeast *Saccharomyces cerevisiae *at seven time points during sporulation using spotted microarrays, and identified seven distinct temporal patterns of induction [22]. 39 representative genes were used to define the model expression profile for each pattern. Based on their properties, the seven patterns are designated as Metabolic, Early I, Early II, Early-Mid, Middle, Mid-Late and Late. The average expression profiles for these seven patterns are presented in Figure 4. The genes in Early I, Early II, Middle, Mid-Late and Late initiates induction of expression at 0.5 h, 2 h, 5 h, 7 h and 9 h, respectively, and sustains expression through the rest of the time course. The expression of metabolic genes is also induced at 0.5 h as in Early I, but decays afterwards. The expression of genes in Early-Mid is induced not only at the 0.5 h and 2 h as in Early genes, but also at 5 h and 7 h, as in the Middle and Mid-Late genes. This data structure made it difficult to separate the Early-Mid genes from others. The direct clustering analyses using *PearsonC *or *Eucli *were not successful.

**Figure 5:**
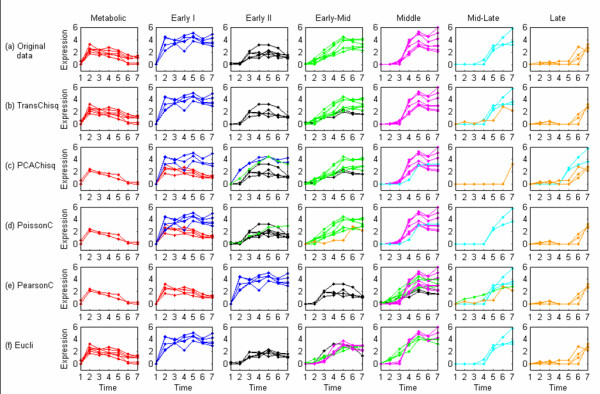
**Clustering results for the yeast sporulation data**. (a) Original expression profiles of the 39 representative genes from 7 functional groups, (b)-(f) Expression profiles of the 7 clusters produced by different clustering algorithms. The x-axis represents different time points of 0h, 0.5 h, 2 h, 5 h, 7 h, 9 h, 11.5 h. The y-axis represents the normalized log-ratio expression levels.

Prior to analyzing the data we substituted the expression ratios that were below zero with zeros as in Figure 5(a). This truncation of negative values simplifies the expression patterns of the 39 representative genes with the key properties in each pattern being intact. The clustering results are summarized in Table 5. We can see that *TransChisq *outperforms other methods: 3, 7, 8, 13, 14 and 17 of the 39 genes are incorrectly clustered by *TransChisq*, *PoissonC*, *Eucli*, *PearsonC*, *PCAChisq *and *Eucli *on rescaled data respectively. *TransChisq *also shows the best adjusted Rand Index. It is interesting to see that the performance of *Eucli *on rescaled data is worse than it is on original data. This suggests that the magnitude information should be critical and cannot be ignored in determining the seven classes. As we have discussed, all methods fail to discern the genes in Early-Mid from the genes in Early I, Early II, Middle, Mid-Late and Late (Figure 5(b)–(f)). Furthermore, *PCAChisq *and *PoissonC *mixed up two different patterns from Metabolic and Early I because of their similar induction time at 0.5 h (Figure 5(c) and 5(d)). *PearsonC *even splits the Metabolic group further into two separate clusters (Figure 5(e)).

**Table 5:** Comparison of the algorithms on the 39 yeast sporulation genes

Algorithm	Number of genes in incorrect clusters	% of genes in incorrect clusters	Adjusted Rand Index
*TransChisq*	3	7.7	0.830
*PCAChisq*	14	35.9	0.527
*PoissonC*	7	18.0	0.675
*PearsonC*	13	33.3	0.483
*Eucli*	8	20.5	0.600
*Eucli *on rescaled data	17	43.6	0.483
*SRC*	NA	NA	0.325

For *PCAChisq*, we tried different combinations of principal components (PCs) to optimize the clustering results. The best result can be reached when the first 5 PCs were used: 3 out of the 39 genes were incorrectly grouped. This optimal result is the same as the one from *TransChisq*. However, in practice, it is not feasible to exhaust all possible combinations of PCs to search for the optimal clustering result.

### Estimating the number of clusters using Gap Statistics

An unsolved issue in *K*-means clustering analysis is how to estimate *K*, the number of clusters. In the recent literature the Gap statistic was found useful [25,26]. The technique of the Gap statistic uses the output of any clustering algorithm to compare the 'between-to-total variance (*R*^2^)' with that expected under an appropriate reference null distribution. A high *R*^2 ^value represents high variability between clusters and high coherence within clusters. Below we sketch how to calculate the Gap statistic: Let *D*_*k *_be the *R*^2 ^measure for the clustering output when the number of clusters is *k*. To derive the reference expected value of *D*_*k*_, the elements within each row of original data are permuted to produce the new matrices with random profile patterns. Assume *B *such matrices are obtained. Then for each matrix, a new *R*^2 ^is calculated based on the original clustering output and the pre-selected similarity measure. The average of these *R*^2^'s, denoted by D¯k
 MathType@MTEF@5@5@+=feaafiart1ev1aaatCvAUfKttLearuWrP9MDH5MBPbIqV92AaeXatLxBI9gBaebbnrfifHhDYfgasaacH8akY=wiFfYdH8Gipec8Eeeu0xXdbba9frFj0=OqFfea0dXdd9vqai=hGuQ8kuc9pgc9s8qqaq=dirpe0xb9q8qiLsFr0=vr0=vr0dc8meaabaqaciaacaGaaeqabaqabeGadaaakeaadaqdaaqaaiabdseaebaadaWgaaWcbaGaem4AaSgabeaaaaa@2F59@, serves as the expectation of *D*_*k*_. With *D*_*k*_and D¯k
 MathType@MTEF@5@5@+=feaafiart1ev1aaatCvAUfKttLearuWrP9MDH5MBPbIqV92AaeXatLxBI9gBaebbnrfifHhDYfgasaacH8akY=wiFfYdH8Gipec8Eeeu0xXdbba9frFj0=OqFfea0dXdd9vqai=hGuQ8kuc9pgc9s8qqaq=dirpe0xb9q8qiLsFr0=vr0=vr0dc8meaabaqaciaacaGaaeqabaqabeGadaaakeaadaqdaaqaaiabdseaebaadaWgaaWcbaGaem4AaSgabeaaaaa@2F59@, the Gap function is defined by

Gap(*k*)= *D*_*k *_- D¯k
 MathType@MTEF@5@5@+=feaafiart1ev1aaatCvAUfKttLearuWrP9MDH5MBPbIqV92AaeXatLxBI9gBaebbnrfifHhDYfgasaacH8akY=wiFfYdH8Gipec8Eeeu0xXdbba9frFj0=OqFfea0dXdd9vqai=hGuQ8kuc9pgc9s8qqaq=dirpe0xb9q8qiLsFr0=vr0=vr0dc8meaabaqaciaacaGaaeqabaqabeGadaaakeaadaqdaaqaaiabdseaebaadaWgaaWcbaGaem4AaSgabeaaaaa@2F59@.

The value of *k *with the largest Gap value will be selected as the optimal number of clusters in that at this *k*, the observed between-to-total variance *R*^2 ^is the most ahead of expected.

**Figure 6:**
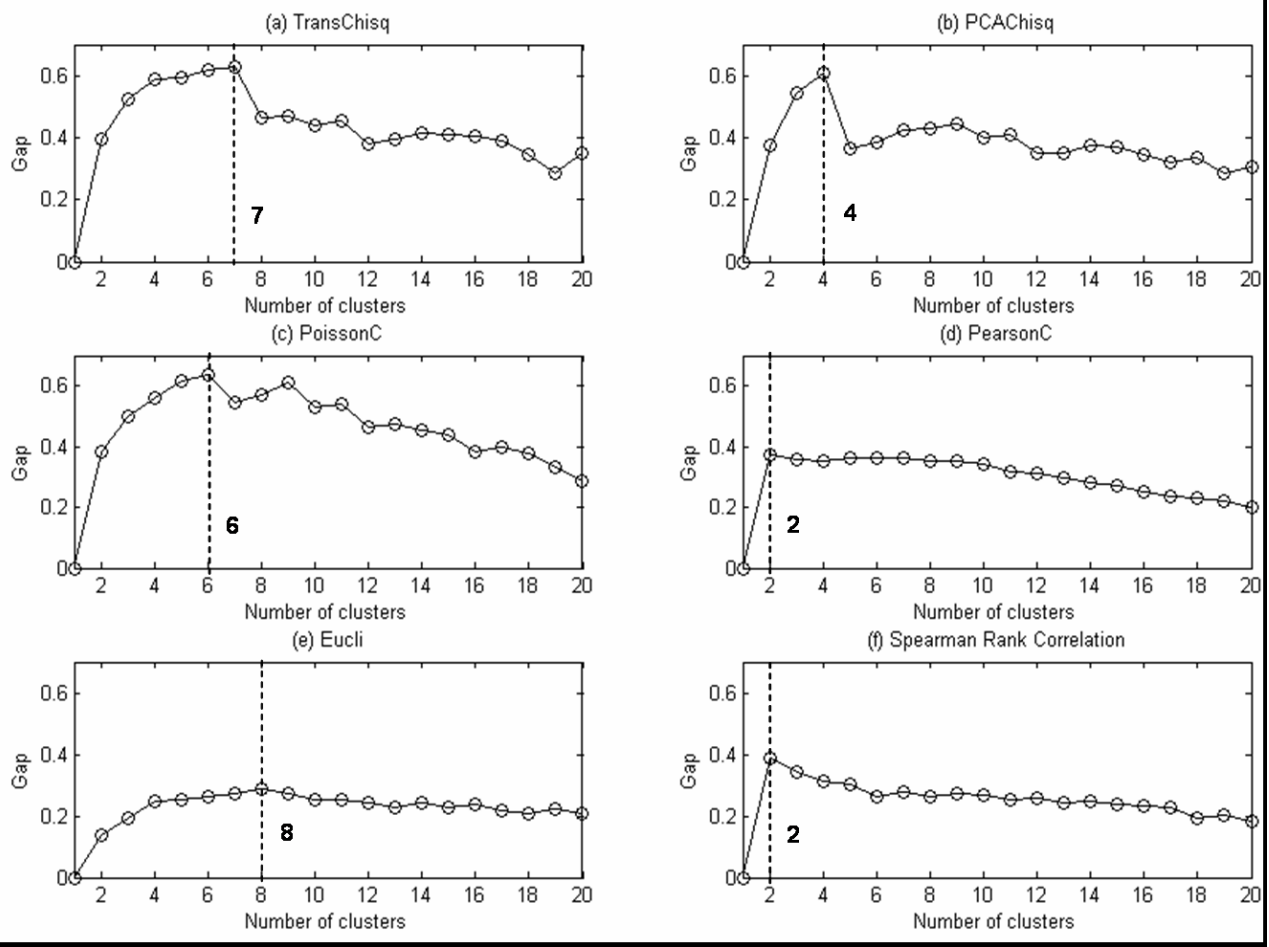
**Gap statistic results on the 39 yeast sporulation genes**. The x-axis represents the number of clusters and the y-axis represents the gap statistics over different number of clusters. In each sub-figure, the x-axis value associated with the largest gap statistic is the optimal selection of the number of clusters under the used similarity measure. From the shown gap curves, only *TransChisq *provides a correct estimate on the true number of clusters, 7.

For comparison, we used different measures including *TransChisq*, *PCAChisq*, *PoissonC*, *Pearson*, *Eucli*, and *SRC *to calculate the Gap statistics for each of the two experimental datasets: microarray yeast sporulation data and mouse retinal SAGE data. For the microarray yeast sporulation data, the Gap values from different measures over different number of clusters are shown in Figure 6. We can see that *TransChisq *shows the maximum Gap value at *k *= 7. In other words, *TransChisq *finds an optimal number of 7 clusters, which agrees with the known functional categorization of the genes. Other measures all produce incorrect estimates of the number of clusters on the same dataset. In a similar analysis of the SAGE data, *TransChisq*, *PCAChisq *and *PoissonC *provide a correct estimate on the number of clusters, 5. *PearsonC*, *Eucli *and *SRC *give an incorrect estimate of 3, 14 and 2 respectively (the gap function curves are not shown here). This study shows that when the number of clusters, *K*, is unknown, the Gap Statistics can be used to estimate *K*, and *TransChisq *is favorable over others on estimating the true number of clusters in both experimental datasets.

## Discussions and conclusions

In this study, we proposed a method, *TransChisq*, to group genes with similar expression shapes. The expression magnitude was considered when measuring the shape similarity. Results from applications to a variety of datasets demonstrated *TransChisq*'s clear advantages over other methods. Furthermore, with the gap statistics, *TransChisq *was also found to be effective in estimating the number of clusters. Regarding the computational efficiency, *TransChisq*, *PCAChisq *and *PoissonC *have similar costs but usually run a few times (2 to 5 times) slower than the *PearsonC *and *Eucli*.

We have embedded different measures in the *K*-means clustering procedure to reveal the important gene expression patterns. In addition to *K*-means, our new measure can also be implemented in other clustering methods, e.g., hierarchical clustering [4], to perform the analysis. In a hierarchical clustering procedure, the distance of any two gene expression profiles can be defined using measure (4) by assuming that two genes form a cluster. A study on the performance of different measures in a hierarchical clustering procedure is in Additional file 2. Our new method also outperforms others when implemented in the hierarchical clustering algorithm.

We view different measures as complementary rather than competing in that each has its advantages. In general, *TransChisq *would be effective when it is necessary to consider the magnitude information in measuring the shape similarity. In clustering analyses of SAGE and microarray data, very often the magnitude information should be taken into account, whereas the shape could be a more critical factor to determine the gene relationship.

Although the proposed method is very promising, it does require further study on possible data transformation schemes when the original data show a more complex structure, or when the clustering purpose is different. We suggest our method could provide new insights to the applications of different data transformations in clustering analysis of gene expression data.

## Methods

The underlying probability model of our new measures was adopted from the work of Cai et al. [20], where two Poisson based measures were proposed for clustering analysis of SAGE data, or more generally, Poisson distributed data. A brief review on this work is presented below, followed by a detailed description of the newly proposed measures.

### PoissonC and PoissonL for clustering analysis of SAGE data

SAGE is one of the effective techniques for comprehensive gene expression profiling. The result of a SAGE experiment, called a SAGE library, is a list of counts of sequenced tags isolated from mRNAs that are randomly sampled from a cell or tissue. As discussed in Man et al. [27], the sampling process for tag extraction is approximately equivalent to randomly taking a bag of colored balls from a big box. This randomness leads to an approximate multinomial distribution for the number of transcripts of different types. Moreover, due to the vast amount of varied types of transcripts in a cell or tissue, the selection probability of a particular type of transcript at each draw should be very small. This suggests that the tag counts of sampled transcripts of each type are approximately Poisson distributed. *PoissonC *and *PoissonL *were developed under this context [20]. The method is summarized below.

Let *Y*_*i*_(*t*) be the count of tag *i *in library *t*, and ***Y***_*i *_= (*Y*_*i*_(1),..., *Y*_*i*_(*T*)) be the vector of counts of tag *i *over a total of *T *libraries. *Y*_*i*_(*t*) is assumed to be Poisson distributed with mean *γ*_*it*_. To model the magnitude and shape of the expression profile separately, Cai et al. [20] further parameterized the Poisson rate as *γ*_*it *_= *λ*_*i*_(*t*)*θ*_*i*_, where *θ*_*i *_is the expected sum of counts of tag *i *over all libraries, and *λ*_*i *_(*t*) is the contribution of tag *i *in library *t *to the sum *θ*_*i *_expressed in percentage. The sum of *λ*_*i*_(*t*) over all libraries equals to 1. So *λ*_*i*_(*t*)*θ*_*i *_redistributes the tag counts according to the expression shape parameter (*λ*_*i*_(*t*)'s) but keeps the sum of counts over libraries constant. The genes with similar *λ*_*i*_(*t*)'s over *t *are considered to be in the same cluster.

For a cluster consisting of tags 1,2,..., *m *with the common shape parameter *λ *= (*λ*(1),..., *λ*(*T*)), the joint likelihood function for ***Y***_1_, ***Y***_2_,...,***Y***_*m *_is

L(λ,θ|Y)∝f(Y1,...,Ym|λ,θ1,...,θm)=∏i=1m∏t=1Texp⁡(−λ(t)θi)(λ(t)θi)Yi(t)Yi(t)!     (1)
 MathType@MTEF@5@5@+=feaafiart1ev1aaatCvAUfKttLearuWrP9MDH5MBPbIqV92AaeXatLxBI9gBaebbnrfifHhDYfgasaacH8akY=wiFfYdH8Gipec8Eeeu0xXdbba9frFj0=OqFfea0dXdd9vqai=hGuQ8kuc9pgc9s8qqaq=dirpe0xb9q8qiLsFr0=vr0=vr0dc8meaabaqaciaacaGaaeqabaqabeGadaaakeaacqWGmbatcqGGOaakiiWacqWF7oaBcqGGSaalcqWF4oqCcqGG8baFieWacqGFzbqwcqGGPaqkcqGHDisTcqWGMbGzcqGGOaakcqGFzbqwdaWgaaWcbaacbeGae0xmaedabeaakiabcYcaSiabc6caUiabc6caUiabc6caUiabcYcaSiab+LfaznaaBaaaleaacqGFTbqBaeqaaOGaeiiFaWNae83UdWMaeiilaWccciGaeWhUde3aaSbaaSqaaiabigdaXaqabaGccqGGSaalcqGGUaGlcqGGUaGlcqGGUaGlcqGGSaalcqaF4oqCdaWgaaWcbaGaemyBa0gabeaakiabcMcaPiabg2da9maarahabaWaaebCaeaadaWcaaqaaiGbcwgaLjabcIha4jabcchaWjabcIcaOiabgkHiTiab8T7aSjabcIcaOiabdsha0jabcMcaPiab8H7aXnaaBaaaleaacqWGPbqAaeqaaOGaeiykaKIaeiikaGIaeW3UdWMaeiikaGIaemiDaqNaeiykaKIaeWhUde3aaSbaaSqaaiabdMgaPbqabaGccqGGPaqkdaahaaWcbeqaaiabdMfaznaaBaaameaacqWGPbqAaeqaaSGaeiikaGIaemiDaqNaeiykaKcaaaGcbaGaemywaK1aaSbaaSqaaiabdMgaPbqabaGccqGGOaakcqWG0baDcqGGPaqkcqGGHaqiaaaaleaacqWG0baDcqGH9aqpcqaIXaqmaeaacqWGubava0Gaey4dIunaaSqaaiabdMgaPjabg2da9iabigdaXaqaaiabd2gaTbqdcqGHpis1aOGaaCzcaiaaxMaadaqadaqaaiabigdaXaGaayjkaiaawMcaaaaa@8B55@

The maximum likelihood estimates of *λ *and *θ*_1_,..., *θ*_*m *_are

θ_i=∑iYi(t), and λ^(t)=∑i=1mYi(t)/∑i=1mθ_i=∑i=1mYi(t)/∑i=1m∑tYi(t).     (2)
 MathType@MTEF@5@5@+=feaafiart1ev1aaatCvAUfKttLearuWrP9MDH5MBPbIqV92AaeXatLxBI9gBaebbnrfifHhDYfgasaacH8akY=wiFfYdH8Gipec8Eeeu0xXdbba9frFj0=OqFfea0dXdd9vqai=hGuQ8kuc9pgc9s8qqaq=dirpe0xb9q8qiLsFr0=vr0=vr0dc8meaabaqaciaacaGaaeqabaqabeGadaaakeaadaqiaaqaaGGaciab=H7aXbGaayPadaWaaSbaaSqaaiabdMgaPbqabaGccqGH9aqpdaaeqbqaaiabdMfaznaaBaaaleaacqWGPbqAaeqaaOGaeiikaGIaemiDaqNaeiykaKcaleaacqWGPbqAaeqaniabggHiLdGccqGGSaalcqqGGaaicqqGHbqycqqGUbGBcqqGKbazcqqGGaaidaWcgaqaaiqb=T7aSzaajaGaeiikaGIaemiDaqNaeiykaKIaeyypa0ZaaabCaeaacqWGzbqwdaWgaaWcbaGaemyAaKgabeaakiabcIcaOiabdsha0jabcMcaPaWcbaGaemyAaKMaeyypa0JaeGymaedabaGaemyBa0ganiabggHiLdaakeaadaaeWbqaamaaHaaabaGae8hUdehacaGLcmaadaWgaaWcbaGaemyAaKgabeaaaeaacqWGPbqAcqGH9aqpcqaIXaqmaeaacqWGTbqBa0GaeyyeIuoaaaGccqGH9aqpdaWcgaqaamaaqahabaGaemywaK1aaSbaaSqaaiabdMgaPbqabaGccqGGOaakcqWG0baDcqGGPaqkaSqaaiabdMgaPjabg2da9iabigdaXaqaaiabd2gaTbqdcqGHris5aaGcbaWaaabCaeaadaaeqbqaaiabdMfaznaaBaaaleaacqWGPbqAaeqaaOGaeiikaGIaemiDaqNaeiykaKcaleaacqWG0baDaeqaniabggHiLdaaleaacqWGPbqAcqGH9aqpcqaIXaqmaeaacqWGTbqBa0GaeyyeIuoaaaGccqGGUaGlcaWLjaGaaCzcamaabmaabaGaeGOmaidacaGLOaGaayzkaaaaaa@82AF@

Formula (2) forms the basis of the following two measures for evaluating how well a particular tag fits in a cluster. One natural measure is to use the log-likelihood function: log *f*(***Y***_*i*_|***λ***, *θ*_*i*_). The larger the log-likelihood is, the more likely the observed counts are generated from the expected Poisson distributions. So for a cluster consisting of tags 1,2,..., *m*, a likelihood based measure is defined as

L=−log⁡f(Y1,...,Ym|λ^,θ^)=∑i=1m∑t=1T(λ^(t)θ^i−Yi(t)log⁡(λ^(t)θ^i)+log⁡(Yi(t)!)).     (3)
 MathType@MTEF@5@5@+=feaafiart1ev1aaatCvAUfKttLearuWrP9MDH5MBPbIqV92AaeXatLxBI9gBaebbnrfifHhDYfgasaacH8akY=wiFfYdH8Gipec8Eeeu0xXdbba9frFj0=OqFfea0dXdd9vqai=hGuQ8kuc9pgc9s8qqaq=dirpe0xb9q8qiLsFr0=vr0=vr0dc8meaabaqaciaacaGaaeqabaqabeGadaaakeaacqWGmbatcqGH9aqpcqGHsislcyGGSbaBcqGGVbWBcqGGNbWzcqWGMbGzcqGGOaakieWacqWFzbqwdaWgaaWcbaacbeGae4xmaedabeaakiabcYcaSiabc6caUiabc6caUiabc6caUiabcYcaSiab=LfaznaaBaaaleaacqWFTbqBaeqaaOGaeiiFaW3aaecaaeaaiiWacqqF7oaBaiaawkWaaiabcYcaSmaaHaaabaGae0hUdehacaGLcmaacqGGPaqkcqGH9aqpdaaeWaqaamaaqadabaGaeiikaGYaaecaaeaaiiGacqaF7oaBaiaawkWaaiabcIcaOiabdsha0jabcMcaPmaaHaaabaGaeWhUdehacaGLcmaadaWgaaWcbaGaemyAaKgabeaakiabgkHiTiabdMfaznaaBaaaleaacqWGPbqAaeqaaOGaeiikaGIaemiDaqNaeiykaKIagiiBaWMaei4Ba8Maei4zaCMaeiikaGYaaecaaeaacqaF7oaBaiaawkWaaiabcIcaOiabdsha0jabcMcaPmaaHaaabaGaeWhUdehacaGLcmaadaWgaaWcbaGaemyAaKgabeaakiabcMcaPiabgUcaRiGbcYgaSjabc+gaVjabcEgaNjabcIcaOiabdMfaznaaBaaaleaacqWGPbqAaeqaaOGaeiikaGIaemiDaqNaeiykaKIaeiyiaeIaeiykaKIaeiykaKcaleaacqWG0baDcqGH9aqpcqaIXaqmaeaacqWGubava0GaeyyeIuoaaSqaaiabdMgaPjabg2da9iabigdaXaqaaiabd2gaTbqdcqGHris5aOGaeiOla4IaaCzcaiaaxMaadaqadaqaaiabiodaZaGaayjkaiaawMcaaaaa@89D1@

The other measure is based on the Chi-square statistic, a well known statistic for evaluating the deviation of the observations from the expected values. It is defined as

D=∑i=1m∑t=1T(Yi(t)−λ^(t)θ^i)2/(λ^(t)θ^i).     (4)
 MathType@MTEF@5@5@+=feaafiart1ev1aaatCvAUfKttLearuWrP9MDH5MBPbIqV92AaeXatLxBI9gBaebbnrfifHhDYfgasaacH8akY=wiFfYdH8Gipec8Eeeu0xXdbba9frFj0=OqFfea0dXdd9vqai=hGuQ8kuc9pgc9s8qqaq=dirpe0xb9q8qiLsFr0=vr0=vr0dc8meaabaqaciaacaGaaeqabaqabeGadaaakeaacqWGebarcqGH9aqpdaaeWaqaamaaqadabaWaaSGbaeaacqGGOaakcqWGzbqwdaWgaaWcbaGaemyAaKgabeaakiabcIcaOiabdsha0jabcMcaPiabgkHiTmaaHaaabaacciGae83UdWgacaGLcmaacqGGOaakcqWG0baDcqGGPaqkdaqiaaqaaiab=H7aXbGaayPadaWaaSbaaSqaaiabdMgaPbqabaGccqGGPaqkdaahaaWcbeqaaiabikdaYaaaaOqaaiabcIcaOmaaHaaabaGae83UdWgacaGLcmaacqGGOaakcqWG0baDcqGGPaqkdaqiaaqaaiab=H7aXbGaayPadaWaaSbaaSqaaiabdMgaPbqabaGccqGGPaqkaaaaleaacqWG0baDcqGH9aqpcqaIXaqmaeaacqWGubava0GaeyyeIuoaaSqaaiabdMgaPjabg2da9iabigdaXaqaaiabd2gaTbqdcqGHris5aOGaeiOla4IaaCzcaiaaxMaadaqadaqaaiabisda0aGaayjkaiaawMcaaaaa@5F7F@

Using Chi-square statistic as a similarity measure, the penalty for the deviation from large expected count is smaller than that for small expected count. It is consistent with the above likelihood-based measure in that the variance of a Poisson variable equals to its mean. In general, the smaller the value of *L *or *D*, the more likely the tags belong to the same cluster. We should also note that the statistics in measure (3) and measure (4) consider both the shape and magnitude information when measuring the cluster dispersion, i.e., the cluster is specified by the shape parameter ***λ***, but the relationship of a tag to a certain cluster is determined by the deviation of observed counts (θ^
 MathType@MTEF@5@5@+=feaafiart1ev1aaatCvAUfKttLearuWrP9MDH5MBPbIqV92AaeXatLxBI9gBaebbnrfifHhDYfgasaacH8akY=wiFfYdH8Gipec8Eeeu0xXdbba9frFj0=OqFfea0dXdd9vqai=hGuQ8kuc9pgc9s8qqaq=dirpe0xb9q8qiLsFr0=vr0=vr0dc8meaabaqaciaacaGaaeqabaqabeGadaaakeaadaqiaaqaaGGaciab=H7aXbGaayPadaaaaa@2F2B@_*i *_λ^
 MathType@MTEF@5@5@+=feaafiart1ev1aaatCvAUfKttLearuWrP9MDH5MBPbIqV92AaeXatLxBI9gBaebbnrfifHhDYfgasaacH8akY=wiFfYdH8Gipec8Eeeu0xXdbba9frFj0=OqFfea0dXdd9vqai=hGuQ8kuc9pgc9s8qqaq=dirpe0xb9q8qiLsFr0=vr0=vr0dc8meaabaqaciaacaGaaeqabaqabeGadaaakeaadaqiaaqaaGGadiab=T7aSbGaayPadaaaaa@2F2A@_*i*_) from the expected values (θ^
 MathType@MTEF@5@5@+=feaafiart1ev1aaatCvAUfKttLearuWrP9MDH5MBPbIqV92AaeXatLxBI9gBaebbnrfifHhDYfgasaacH8akY=wiFfYdH8Gipec8Eeeu0xXdbba9frFj0=OqFfea0dXdd9vqai=hGuQ8kuc9pgc9s8qqaq=dirpe0xb9q8qiLsFr0=vr0=vr0dc8meaabaqaciaacaGaaeqabaqabeGadaaakeaadaqiaaqaaGGaciab=H7aXbGaayPadaaaaa@2F2B@_*i *_*λ*). Here λ^
 MathType@MTEF@5@5@+=feaafiart1ev1aaatCvAUfKttLearuWrP9MDH5MBPbIqV92AaeXatLxBI9gBaebbnrfifHhDYfgasaacH8akY=wiFfYdH8Gipec8Eeeu0xXdbba9frFj0=OqFfea0dXdd9vqai=hGuQ8kuc9pgc9s8qqaq=dirpe0xb9q8qiLsFr0=vr0=vr0dc8meaabaqaciaacaGaaeqabaqabeGadaaakeaadaqiaaqaaGGadiab=T7aSbGaayPadaaaaa@2F2A@_*i *_is the estimated profile shape of tag *i *(λ^
 MathType@MTEF@5@5@+=feaafiart1ev1aaatCvAUfKttLearuWrP9MDH5MBPbIqV92AaeXatLxBI9gBaebbnrfifHhDYfgasaacH8akY=wiFfYdH8Gipec8Eeeu0xXdbba9frFj0=OqFfea0dXdd9vqai=hGuQ8kuc9pgc9s8qqaq=dirpe0xb9q8qiLsFr0=vr0=vr0dc8meaabaqaciaacaGaaeqabaqabeGadaaakeaadaqiaaqaaGGadiab=T7aSbGaayPadaaaaa@2F2A@_*i *_= (λ^
 MathType@MTEF@5@5@+=feaafiart1ev1aaatCvAUfKttLearuWrP9MDH5MBPbIqV92AaeXatLxBI9gBaebbnrfifHhDYfgasaacH8akY=wiFfYdH8Gipec8Eeeu0xXdbba9frFj0=OqFfea0dXdd9vqai=hGuQ8kuc9pgc9s8qqaq=dirpe0xb9q8qiLsFr0=vr0=vr0dc8meaabaqaciaacaGaaeqabaqabeGadaaakeaadaqiaaqaaGGaciab=T7aSbGaayPadaaaaa@2F29@_*i *_(1),...,λ^
 MathType@MTEF@5@5@+=feaafiart1ev1aaatCvAUfKttLearuWrP9MDH5MBPbIqV92AaeXatLxBI9gBaebbnrfifHhDYfgasaacH8akY=wiFfYdH8Gipec8Eeeu0xXdbba9frFj0=OqFfea0dXdd9vqai=hGuQ8kuc9pgc9s8qqaq=dirpe0xb9q8qiLsFr0=vr0=vr0dc8meaabaqaciaacaGaaeqabaqabeGadaaakeaadaqiaaqaaGGaciab=T7aSbGaayPadaaaaa@2F29@_*i *_(*T*)) and λ^i(t)=Yi(t)/∑tYi(t)=Yi(t)/θ^i
 MathType@MTEF@5@5@+=feaafiart1ev1aaatCvAUfKttLearuWrP9MDH5MBPbIqV92AaeXatLxBI9gBaebbnrfifHhDYfgasaacH8akY=wiFfYdH8Gipec8Eeeu0xXdbba9frFj0=OqFfea0dXdd9vqai=hGuQ8kuc9pgc9s8qqaq=dirpe0xb9q8qiLsFr0=vr0=vr0dc8meaabaqaciaacaGaaeqabaqabeGadaaakeaadaWcgaqaamaaHaaabaacciGae83UdWgacaGLcmaadaWgaaWcbaGaemyAaKgabeaakiabcIcaOiabdsha0jabcMcaPiabg2da9iabdMfaznaaBaaaleaacqWGPbqAaeqaaOGaeiikaGIaemiDaqNaeiykaKcabaWaaabeaeaacqWGzbqwdaWgaaWcbaGaemyAaKgabeaaaeaacqWG0baDaeqaniabggHiLdaaaOGaeiikaGIaemiDaqNaeiykaKIaeyypa0ZaaSGbaeaacqWGzbqwdaWgaaWcbaGaemyAaKgabeaakiabcIcaOiabdsha0jabcMcaPaqaamaaHaaabaGae8hUdehacaGLcmaadaWgaaWcbaGaemyAaKgabeaaaaaaaa@4F25@). A measure that ignores magnitude would take the difference between λ^
 MathType@MTEF@5@5@+=feaafiart1ev1aaatCvAUfKttLearuWrP9MDH5MBPbIqV92AaeXatLxBI9gBaebbnrfifHhDYfgasaacH8akY=wiFfYdH8Gipec8Eeeu0xXdbba9frFj0=OqFfea0dXdd9vqai=hGuQ8kuc9pgc9s8qqaq=dirpe0xb9q8qiLsFr0=vr0=vr0dc8meaabaqaciaacaGaaeqabaqabeGadaaakeaadaqiaaqaaGGadiab=T7aSbGaayPadaaaaa@2F2A@_*i *_and ***λ*** directly.

Cai et al. [20] have employed the above measures into a *K*-means clustering algorithm to perform clustering analysis. *K*-means clustering procedure [5] generates clusters by assigning each object to one of *K *clusters so as to minimize a measure of dispersion within the clusters. The algorithm is outlined below:

1. All SAGE tags are assigned randomly to *K *sets. Estimate initial parameters θi(0)
 MathType@MTEF@5@5@+=feaafiart1ev1aaatCvAUfKttLearuWrP9MDH5MBPbIqV92AaeXatLxBI9gBaebbnrfifHhDYfgasaacH8akY=wiFfYdH8Gipec8Eeeu0xXdbba9frFj0=OqFfea0dXdd9vqai=hGuQ8kuc9pgc9s8qqaq=dirpe0xb9q8qiLsFr0=vr0=vr0dc8meaabaqaciaacaGaaeqabaqabeGadaaakeaaiiGacqWF4oqCdaqhaaWcbaGaemyAaKgabaGaeiikaGIaeGimaaJaeiykaKcaaaaa@3291@ and λk(0)=(λk(0)(1),...,λk0(T))
 MathType@MTEF@5@5@+=feaafiart1ev1aaatCvAUfKttLearuWrP9MDH5MBPbIqV92AaeXatLxBI9gBaebbnrfifHhDYfgasaacH8akY=wiFfYdH8Gipec8Eeeu0xXdbba9frFj0=OqFfea0dXdd9vqai=hGuQ8kuc9pgc9s8qqaq=dirpe0xb9q8qiLsFr0=vr0=vr0dc8meaabaqaciaacaGaaeqabaqabeGadaaakeaaiiWacqWF7oaBdaqhaaWcbaGaem4AaSgabaGaeiikaGIaeGimaaJaeiykaKcaaOGaeyypa0JaeiikaGccciGae43UdW2aa0baaSqaaiabdUgaRbqaaiabcIcaOiabicdaWiabcMcaPaaakiabcIcaOiabigdaXiabcMcaPiabcYcaSiabc6caUiabc6caUiabc6caUiabcYcaSiab+T7aSnaaDaaaleaacqWGRbWAaeaacqaIWaamaaGccqGGOaakcqWGubavcqGGPaqkcqGGPaqkaaa@4969@ for each tag and each cluster by formula (2).

2. In the (b+1)th iteration, assign each tag *i *to the cluster with minimum deviation from the expected model. The deviation is measured by either Li,k(b)=−log⁡f(Yi|λk(b),θi(b))
 MathType@MTEF@5@5@+=feaafiart1ev1aaatCvAUfKttLearuWrP9MDH5MBPbIqV92AaeXatLxBI9gBaebbnrfifHhDYfgasaacH8akY=wiFfYdH8Gipec8Eeeu0xXdbba9frFj0=OqFfea0dXdd9vqai=hGuQ8kuc9pgc9s8qqaq=dirpe0xb9q8qiLsFr0=vr0=vr0dc8meaabaqaciaacaGaaeqabaqabeGadaaakeaacqWGmbatdaqhaaWcbaGaemyAaKMaeiilaWIaem4AaSgabaGaeiikaGIaemOyaiMaeiykaKcaaOGaeyypa0JaeyOeI0IagiiBaWMaei4Ba8Maei4zaCMaemOzayMaeiikaGccbmGae8xwaK1aaSbaaSqaaiabdMgaPbqabaGccqGG8baFiiWacqGF7oaBdaqhaaWcbaGaem4AaSgabaGaeiikaGIaemOyaiMaeiykaKcaaOGaeiilaWccciGae0hUde3aa0baaSqaaiabdMgaPbqaaiabcIcaOiabdkgaIjabcMcaPaaakiabcMcaPaaa@4F85@ or Di,k(b)=∑t(Yi(t)−λk(b)(t)θi(b))2/(λk(b)(t)θi(b))
 MathType@MTEF@5@5@+=feaafiart1ev1aaatCvAUfKttLearuWrP9MDH5MBPbIqV92AaeXatLxBI9gBaebbnrfifHhDYfgasaacH8akY=wiFfYdH8Gipec8Eeeu0xXdbba9frFj0=OqFfea0dXdd9vqai=hGuQ8kuc9pgc9s8qqaq=dirpe0xb9q8qiLsFr0=vr0=vr0dc8meaabaqaciaacaGaaeqabaqabeGadaaakeaacqWGebardaqhaaWcbaGaemyAaKMaeiilaWIaem4AaSgabaGaeiikaGIaemOyaiMaeiykaKcaaOGaeyypa0ZaaabeaeaadaWcgaqaamaabmaabaGaemywaK1aaSbaaSqaaiabdMgaPbqabaGccqGGOaakcqWG0baDcqGGPaqkcqGHsisliiGacqWF7oaBdaqhaaWcbaGaem4AaSgabaGaeiikaGIaemOyaiMaeiykaKcaaOGaeiikaGIaemiDaqNaeiykaKIae8hUde3aa0baaSqaaiabdMgaPbqaaiabcIcaOiabdkgaIjabcMcaPaaaaOGaayjkaiaawMcaamaaCaaaleqabaGaeGOmaidaaaGcbaGaeiikaGIae83UdW2aa0baaSqaaiabdUgaRbqaaiabcIcaOiabdkgaIjabcMcaPaaakiabcIcaOiabdsha0jabcMcaPiab=H7aXnaaDaaaleaacqWGPbqAaeaacqGGOaakcqWGIbGycqGGPaqkaaGccqGGPaqkaaaaleaacqWG0baDaeqaniabggHiLdaaaa@639B@.

3. Set new cluster centers λk(b+1)
 MathType@MTEF@5@5@+=feaafiart1ev1aaatCvAUfKttLearuWrP9MDH5MBPbIqV92AaeXatLxBI9gBaebbnrfifHhDYfgasaacH8akY=wiFfYdH8Gipec8Eeeu0xXdbba9frFj0=OqFfea0dXdd9vqai=hGuQ8kuc9pgc9s8qqaq=dirpe0xb9q8qiLsFr0=vr0=vr0dc8meaabaqaciaacaGaaeqabaqabeGadaaakeaaiiWacqWF7oaBdaqhaaWcbaGaem4AaSgabaGaeiikaGIaemOyaiMaey4kaSIaeGymaeJaeiykaKcaaaaa@34C5@ by formula (2).

4. Repeat step 2 till convergence.

Let *c*(*i*) denote the index of the cluster that tag *i *is assigned to. The above algorithm aims to minimize the within-cluster dispersion ∑_*i*_*L*_*i,c(i) *_or ∑_*i*_*D*_*i,c(i)*_. The algorithm using measure *L *is called *PoissonL*, and the algorithm using measure *D *is called *PoissonC*. *PoissonL *and *PoissonC *perform similarly in applications. But *PoissonC *is more practical in terms of running time. So we use *PoissonC *for comparison in this paper.

*PoissonC *is designed to group the objects by their departure from the expected Poisson distributions. The success of *PoissonC *has been shown in applications [20,21]. However, if the clustering purpose is slightly different, some modification on *PoissonC *may be necessary. For instance, if the shape difference should be more emphasized in determining the relationship, the *direction of departure *of observed from expected may/should also be considered. As an example, we consider an expression vector ***Y ***= (15, 30, 15) and its relationship with two clusters with shape specified by *λ*_1 _= (1/12,5/6,1/12) and *λ*_2 _= (5/12, 1/6, 5/12) respectively. The expectation of ***Y ***in cluster 1 is YE1
 MathType@MTEF@5@5@+=feaafiart1ev1aaatCvAUfKttLearuWrP9MDH5MBPbIqV92AaeXatLxBI9gBaebbnrfifHhDYfgasaacH8akY=wiFfYdH8Gipec8Eeeu0xXdbba9frFj0=OqFfea0dXdd9vqai=hGuQ8kuc9pgc9s8qqaq=dirpe0xb9q8qiLsFr0=vr0=vr0dc8meaabaqaciaacaGaaeqabaqabeGadaaakeaaieWacqWFzbqwdaqhaaWcbaGaemyraueabaGaeGymaedaaaaa@301F@ = (5, 50, 5), and in cluster 2, it is YE2
 MathType@MTEF@5@5@+=feaafiart1ev1aaatCvAUfKttLearuWrP9MDH5MBPbIqV92AaeXatLxBI9gBaebbnrfifHhDYfgasaacH8akY=wiFfYdH8Gipec8Eeeu0xXdbba9frFj0=OqFfea0dXdd9vqai=hGuQ8kuc9pgc9s8qqaq=dirpe0xb9q8qiLsFr0=vr0=vr0dc8meaabaqaciaacaGaaeqabaqabeGadaaakeaaieWacqWFzbqwdaqhaaWcbaGaemyraueabaGaeGOmaidaaaaa@3021@ = (25, 10, 25). If more emphasis should be put on the shape change in determining the relationship, ***Y ***would be expected to be closer to the first cluster because of the large value observed on the middle component in both ***Y ***and YE1
 MathType@MTEF@5@5@+=feaafiart1ev1aaatCvAUfKttLearuWrP9MDH5MBPbIqV92AaeXatLxBI9gBaebbnrfifHhDYfgasaacH8akY=wiFfYdH8Gipec8Eeeu0xXdbba9frFj0=OqFfea0dXdd9vqai=hGuQ8kuc9pgc9s8qqaq=dirpe0xb9q8qiLsFr0=vr0=vr0dc8meaabaqaciaacaGaaeqabaqabeGadaaakeaaieWacqWFzbqwdaqhaaWcbaGaemyraueabaGaeGymaedaaaaa@301F@. *PoissonC*, however, determines that ***Y ***has the same distance to YE1
 MathType@MTEF@5@5@+=feaafiart1ev1aaatCvAUfKttLearuWrP9MDH5MBPbIqV92AaeXatLxBI9gBaebbnrfifHhDYfgasaacH8akY=wiFfYdH8Gipec8Eeeu0xXdbba9frFj0=OqFfea0dXdd9vqai=hGuQ8kuc9pgc9s8qqaq=dirpe0xb9q8qiLsFr0=vr0=vr0dc8meaabaqaciaacaGaaeqabaqabeGadaaakeaaieWacqWFzbqwdaqhaaWcbaGaemyraueabaGaeGymaedaaaaa@301F@ and YE2
 MathType@MTEF@5@5@+=feaafiart1ev1aaatCvAUfKttLearuWrP9MDH5MBPbIqV92AaeXatLxBI9gBaebbnrfifHhDYfgasaacH8akY=wiFfYdH8Gipec8Eeeu0xXdbba9frFj0=OqFfea0dXdd9vqai=hGuQ8kuc9pgc9s8qqaq=dirpe0xb9q8qiLsFr0=vr0=vr0dc8meaabaqaciaacaGaaeqabaqabeGadaaakeaaieWacqWFzbqwdaqhaaWcbaGaemyraueabaGaeGOmaidaaaaa@3021@ (by the measure (4), the distance between ***Y ***and YE1
 MathType@MTEF@5@5@+=feaafiart1ev1aaatCvAUfKttLearuWrP9MDH5MBPbIqV92AaeXatLxBI9gBaebbnrfifHhDYfgasaacH8akY=wiFfYdH8Gipec8Eeeu0xXdbba9frFj0=OqFfea0dXdd9vqai=hGuQ8kuc9pgc9s8qqaq=dirpe0xb9q8qiLsFr0=vr0=vr0dc8meaabaqaciaacaGaaeqabaqabeGadaaakeaaieWacqWFzbqwdaqhaaWcbaGaemyraueabaGaeGymaedaaaaa@301F@ is 48, so is the distance between ***Y ***and YE2
 MathType@MTEF@5@5@+=feaafiart1ev1aaatCvAUfKttLearuWrP9MDH5MBPbIqV92AaeXatLxBI9gBaebbnrfifHhDYfgasaacH8akY=wiFfYdH8Gipec8Eeeu0xXdbba9frFj0=OqFfea0dXdd9vqai=hGuQ8kuc9pgc9s8qqaq=dirpe0xb9q8qiLsFr0=vr0=vr0dc8meaabaqaciaacaGaaeqabaqabeGadaaakeaaieWacqWFzbqwdaqhaaWcbaGaemyraueabaGaeGOmaidaaaaa@3021@). *PoissonC *ignores the *direction of departure*. To address this omission we propose to emphasize the profile shape through suitable data transformations, and to define a distance measure in the transformed space. The construction of a proper feature space under a certain clustering purpose is essential to define an effective distance or similarity measure.

### Proposed distance measures (I): TransChisq

A simple yet natural data transformation to emphasize the expression shape is to consider the mutual differences of the original vector components. Given a gene with expression profile ***Y***_*i *_= (*Y*_*i*_(1),..., *Y*_*i*_(*T*)) the transformed vector ***Z***_*i *_is of dimension *T*(*T*-1)/2 with components in the form of *Y*_*i*_(*t*_1_)-*Y*_*i*_(*t*_2_) for *t*_1 _= 1,..., *T*-1 and *t*_2 _= (*t*_1 _+ 1),..., *T*.

According to the Poisson model in the previous section, *E*(*Y*_*i*_(*t*_1_)-*Y*_*i*_(*t*_2_)) = (*λ*_*i*_(*t*_1_)-*λ*_*i*_(*t*_2_))*θ*_*i *_and *Var*(*Y*_*i*_(*t*_1_)-*Y*_*i*_(*t*_2_)) = (*λ*_*i*_(*t*_1_)+*λ*_*i*_(*t*_2_))*θ*_*i*_. For a cluster consisting of tags l, 2,..., *m*, we can define the following statistic to measure the cluster dispersion:

Strans=∑i∑t1,t2((Yi(t1)−Yi(t2))−E(Yi(t1)−Yi(t2)))2/Var(Yi(t1)−Yi(t2))=∑i∑t1,t2((Yi(t1)−Yi(t2))−(λ^(t1)θ^i−λ^(t2)θ^i))2/(λ^(t1)θ^i+λ^(t2)θ^i),     (5)
 MathType@MTEF@5@5@+=feaafiart1ev1aaatCvAUfKttLearuWrP9MDH5MBPbIqV92AaeXatLxBI9gBaebbnrfifHhDYfgasaacH8akY=wiFfYdH8Gipec8Eeeu0xXdbba9frFj0=OqFfea0dXdd9vqai=hGuQ8kuc9pgc9s8qqaq=dirpe0xb9q8qiLsFr0=vr0=vr0dc8meaabaqaciaacaGaaeqabaqabeGadaaakeaafaqadeGabaaabaGaem4uam1aaSbaaSqaaiabdsha0jabdkhaYjabdggaHjabd6gaUjabdohaZbqabaGccqGH9aqpdaWcgaqaamaaqafabaWaaabuaeaadaqadaqaamaabmaabaGaemywaK1aaSbaaSqaaiabdMgaPbqabaGcdaqadaqaaiabdsha0naaBaaaleaacqaIXaqmaeqaaaGccaGLOaGaayzkaaGaeyOeI0IaemywaK1aaSbaaSqaaiabdMgaPbqabaGcdaqadaqaaiabdsha0naaBaaaleaacqaIYaGmaeqaaaGccaGLOaGaayzkaaaacaGLOaGaayzkaaGaeyOeI0Iaemyrau0aaeWaaeaacqWGzbqwdaWgaaWcbaGaemyAaKgabeaakmaabmaabaGaemiDaq3aaSbaaSqaaiabigdaXaqabaaakiaawIcacaGLPaaacqGHsislcqWGzbqwdaWgaaWcbaGaemyAaKgabeaakmaabmaabaGaemiDaq3aaSbaaSqaaiabikdaYaqabaaakiaawIcacaGLPaaaaiaawIcacaGLPaaaaiaawIcacaGLPaaadaahaaWcbeqaaiabikdaYaaaaeaacqWG0baDdaWgaaadbaGaeGymaedabeaaliabcYcaSiabdsha0naaBaaameaacqaIYaGmaeqaaaWcbeqdcqGHris5aaWcbaGaemyAaKgabeqdcqGHris5aaGcbaGaemOvayLaemyyaeMaemOCai3aaeWaaeaacqWGzbqwdaWgaaWcbaGaemyAaKgabeaakmaabmaabaGaemiDaq3aaSbaaSqaaiabigdaXaqabaaakiaawIcacaGLPaaacqGHsislcqWGzbqwdaWgaaWcbaGaemyAaKgabeaakmaabmaabaGaemiDaq3aaSbaaSqaaiabikdaYaqabaaakiaawIcacaGLPaaaaiaawIcacaGLPaaaaaaabaWaaSGbaeaacqGH9aqpdaaeqbqaamaaqafabaWaaeWaaeaadaqadaqaaiabdMfaznaaBaaaleaacqWGPbqAaeqaaOWaaeWaaeaacqWG0baDdaWgaaWcbaGaeGymaedabeaaaOGaayjkaiaawMcaaiabgkHiTiabdMfaznaaBaaaleaacqWGPbqAaeqaaOWaaeWaaeaacqWG0baDdaWgaaWcbaGaeGOmaidabeaaaOGaayjkaiaawMcaaaGaayjkaiaawMcaaiabgkHiTmaabmaabaWaaecaaeaaiiGacqWF7oaBaiaawkWaamaabmaabaGaemiDaq3aaSbaaSqaaiabigdaXaqabaaakiaawIcacaGLPaaadaqiaaqaaiab=H7aXbGaayPadaWaaSbaaSqaaiabdMgaPbqabaGccqGHsisldaqiaaqaaiab=T7aSbGaayPadaWaaeWaaeaacqWG0baDdaWgaaWcbaGaeGOmaidabeaaaOGaayjkaiaawMcaamaaHaaabaGae8hUdehacaGLcmaadaWgaaWcbaGaemyAaKgabeaaaOGaayjkaiaawMcaaaGaayjkaiaawMcaamaaCaaaleqabaGaeGOmaidaaaqaaiabdsha0naaBaaameaacqaIXaqmaeqaaSGaeiilaWIaemiDaq3aaSbaaWqaaiabikdaYaqabaaaleqaniabggHiLdaaleaacqWGPbqAaeqaniabggHiLdaakeaadaqadaqaamaaHaaabaGae83UdWgacaGLcmaadaqadaqaaiabdsha0naaBaaaleaacqaIXaqmaeqaaaGccaGLOaGaayzkaaWaaecaaeaacqWF4oqCaiaawkWaamaaBaaaleaacqWGPbqAaeqaaOGaey4kaSYaaecaaeaacqWF7oaBaiaawkWaamaabmaabaGaemiDaq3aaSbaaSqaaiabikdaYaqabaaakiaawIcacaGLPaaadaqiaaqaaiab=H7aXbGaayPadaWaaSbaaSqaaiabdMgaPbqabaaakiaawIcacaGLPaaaaaGaeiilaWcaaiaaxMaacaWLjaWaaeWaaeaacqaI1aqnaiaawIcacaGLPaaaaaa@D011@

where λ^
 MathType@MTEF@5@5@+=feaafiart1ev1aaatCvAUfKttLearuWrP9MDH5MBPbIqV92AaeXatLxBI9gBaebbnrfifHhDYfgasaacH8akY=wiFfYdH8Gipec8Eeeu0xXdbba9frFj0=OqFfea0dXdd9vqai=hGuQ8kuc9pgc9s8qqaq=dirpe0xb9q8qiLsFr0=vr0=vr0dc8meaabaqaciaacaGaaeqabaqabeGadaaakeaadaqiaaqaaGGaciab=T7aSbGaayPadaaaaa@2F29@(*t*) and θ^
 MathType@MTEF@5@5@+=feaafiart1ev1aaatCvAUfKttLearuWrP9MDH5MBPbIqV92AaeXatLxBI9gBaebbnrfifHhDYfgasaacH8akY=wiFfYdH8Gipec8Eeeu0xXdbba9frFj0=OqFfea0dXdd9vqai=hGuQ8kuc9pgc9s8qqaq=dirpe0xb9q8qiLsFr0=vr0=vr0dc8meaabaqaciaacaGaaeqabaqabeGadaaakeaadaqiaaqaaGGaciab=H7aXbGaayPadaaaaa@2F2B@_*i *_can be estimated by formula (2). We call the modified *K*-means algorithm with this measure *TransChisq*. Applying it to the toy example in the previous section, *TransChisq *determines that ***Y ***is closer to YE1
 MathType@MTEF@5@5@+=feaafiart1ev1aaatCvAUfKttLearuWrP9MDH5MBPbIqV92AaeXatLxBI9gBaebbnrfifHhDYfgasaacH8akY=wiFfYdH8Gipec8Eeeu0xXdbba9frFj0=OqFfea0dXdd9vqai=hGuQ8kuc9pgc9s8qqaq=dirpe0xb9q8qiLsFr0=vr0=vr0dc8meaabaqaciaacaGaaeqabaqabeGadaaakeaaieWacqWFzbqwdaqhaaWcbaGaemyraueabaGaeGymaedaaaaa@301F@ as we expected.

To better understand the effects of the proposed data transformation, we performed a simple simulation study and presented the results in Additional file 3.

### Proposed distance measures (II): a parametric-covariance-matrix-based measure

Now we consider a data transformation determined by a parametric covariance matrix:

**R **= cov(**X**) = (*γ*_*ij*_)_*i,j *= 1,..., *T*_, with *γ*_*ij *_= *α *> 0 if *i *= *j *and *γ*_*ij *_= *β *if *i *≠ *j*,

where **X **is the data matrix with *n *observations on the rows and *T *variables on the columns, and **R **is the covariance matrix of the *T *variables. The matrix **R **in this form implies that the variables have identical variances and covariances with each other. These properties are biologically reasonable in that normalized arrays have identical distributions, hence equal variances. Also all pairs of variables would exhibit equal covariance (or un-correlated when *β *= 0) if each component had been equally important (or independent) to determine a class.

A data transformation can be defined through the eigenspace of **R**. One set of column orthonormal eigenvectors, denoted by **e**_1_,**e**_2_,...,**e**_*T*_, is presented in Additional file 4. Given a gene expression profile ***Y***_*i *_= (*Y*_*i*_(1),..., *Y*_*i*_(*T*)), a transformation based on **R **is

***Z***_*i *_= (*Z*_*i*1_,..., *Z*_*i*T_) = ***Y***_*i *_(**e**_1 _**e**_2_...**e**_*T*_).

A convenient property of this transformation is that each component has a clear meaning: with **e**_1 _= [1/T
 MathType@MTEF@5@5@+=feaafiart1ev1aaatCvAUfKttLearuWrP9MDH5MBPbIqV92AaeXatLxBI9gBaebbnrfifHhDYfgasaacH8akY=wiFfYdH8Gipec8Eeeu0xXdbba9frFj0=OqFfea0dXdd9vqai=hGuQ8kuc9pgc9s8qqaq=dirpe0xb9q8qiLsFr0=vr0=vr0dc8meaabaqaciaacaGaaeqabaqabeGadaaakeaadaGcaaqaaiabdsfaubWcbeaaaaa@2DF8@,...,1/T
 MathType@MTEF@5@5@+=feaafiart1ev1aaatCvAUfKttLearuWrP9MDH5MBPbIqV92AaeXatLxBI9gBaebbnrfifHhDYfgasaacH8akY=wiFfYdH8Gipec8Eeeu0xXdbba9frFj0=OqFfea0dXdd9vqai=hGuQ8kuc9pgc9s8qqaq=dirpe0xb9q8qiLsFr0=vr0=vr0dc8meaabaqaciaacaGaaeqabaqabeGadaaakeaadaGcaaqaaiabdsfaubWcbeaaaaa@2DF8@]^T^, **e**_2 _= [1/2
 MathType@MTEF@5@5@+=feaafiart1ev1aaatCvAUfKttLearuWrP9MDH5MBPbIqV92AaeXatLxBI9gBaebbnrfifHhDYfgasaacH8akY=wiFfYdH8Gipec8Eeeu0xXdbba9frFj0=OqFfea0dXdd9vqai=hGuQ8kuc9pgc9s8qqaq=dirpe0xb9q8qiLsFr0=vr0=vr0dc8meaabaqaciaacaGaaeqabaqabeGadaaakeaadaGcaaqaaiabikdaYaWcbeaaaaa@2DB9@, -1/2
 MathType@MTEF@5@5@+=feaafiart1ev1aaatCvAUfKttLearuWrP9MDH5MBPbIqV92AaeXatLxBI9gBaebbnrfifHhDYfgasaacH8akY=wiFfYdH8Gipec8Eeeu0xXdbba9frFj0=OqFfea0dXdd9vqai=hGuQ8kuc9pgc9s8qqaq=dirpe0xb9q8qiLsFr0=vr0=vr0dc8meaabaqaciaacaGaaeqabaqabeGadaaakeaadaGcaaqaaiabikdaYaWcbeaaaaa@2DB9@,0,...,0]^T ^and **e**_3 _= [1/6
 MathType@MTEF@5@5@+=feaafiart1ev1aaatCvAUfKttLearuWrP9MDH5MBPbIqV92AaeXatLxBI9gBaebbnrfifHhDYfgasaacH8akY=wiFfYdH8Gipec8Eeeu0xXdbba9frFj0=OqFfea0dXdd9vqai=hGuQ8kuc9pgc9s8qqaq=dirpe0xb9q8qiLsFr0=vr0=vr0dc8meaabaqaciaacaGaaeqabaqabeGadaaakeaadaGcaaqaaiabiAda2aWcbeaaaaa@2DC1@,1/6
 MathType@MTEF@5@5@+=feaafiart1ev1aaatCvAUfKttLearuWrP9MDH5MBPbIqV92AaeXatLxBI9gBaebbnrfifHhDYfgasaacH8akY=wiFfYdH8Gipec8Eeeu0xXdbba9frFj0=OqFfea0dXdd9vqai=hGuQ8kuc9pgc9s8qqaq=dirpe0xb9q8qiLsFr0=vr0=vr0dc8meaabaqaciaacaGaaeqabaqabeGadaaakeaadaGcaaqaaiabiAda2aWcbeaaaaa@2DC1@,-2/6
 MathType@MTEF@5@5@+=feaafiart1ev1aaatCvAUfKttLearuWrP9MDH5MBPbIqV92AaeXatLxBI9gBaebbnrfifHhDYfgasaacH8akY=wiFfYdH8Gipec8Eeeu0xXdbba9frFj0=OqFfea0dXdd9vqai=hGuQ8kuc9pgc9s8qqaq=dirpe0xb9q8qiLsFr0=vr0=vr0dc8meaabaqaciaacaGaaeqabaqabeGadaaakeaadaGcaaqaaiabiAda2aWcbeaaaaa@2DC1@,0,...,0]^T^, for a profile ***Y ***= (*Y*_1_,..., *Y*_T_), the component associated with **e**_1 _is ***Y*e**_1 _= (*Y*_1 _+ *Y*_2_+...+*Y*_T_)/T
 MathType@MTEF@5@5@+=feaafiart1ev1aaatCvAUfKttLearuWrP9MDH5MBPbIqV92AaeXatLxBI9gBaebbnrfifHhDYfgasaacH8akY=wiFfYdH8Gipec8Eeeu0xXdbba9frFj0=OqFfea0dXdd9vqai=hGuQ8kuc9pgc9s8qqaq=dirpe0xb9q8qiLsFr0=vr0=vr0dc8meaabaqaciaacaGaaeqabaqabeGadaaakeaadaGcaaqaaiabdsfaubWcbeaaaaa@2DF8@, which reflects the general expression level; the component associated with **e**_2 _is ***Y*e**_2 _= (*Y*_1_-*Y*_2_)/2
 MathType@MTEF@5@5@+=feaafiart1ev1aaatCvAUfKttLearuWrP9MDH5MBPbIqV92AaeXatLxBI9gBaebbnrfifHhDYfgasaacH8akY=wiFfYdH8Gipec8Eeeu0xXdbba9frFj0=OqFfea0dXdd9vqai=hGuQ8kuc9pgc9s8qqaq=dirpe0xb9q8qiLsFr0=vr0=vr0dc8meaabaqaciaacaGaaeqabaqabeGadaaakeaadaGcaaqaaiabikdaYaWcbeaaaaa@2DB9@, which reflects the difference between *Y*_1 _and *Y*_2_; the component associated with **e**_3 _is ***Y*e**_3 _= (*Y*_1_+*Y*_2_-2*Y*_3_)/6
 MathType@MTEF@5@5@+=feaafiart1ev1aaatCvAUfKttLearuWrP9MDH5MBPbIqV92AaeXatLxBI9gBaebbnrfifHhDYfgasaacH8akY=wiFfYdH8Gipec8Eeeu0xXdbba9frFj0=OqFfea0dXdd9vqai=hGuQ8kuc9pgc9s8qqaq=dirpe0xb9q8qiLsFr0=vr0=vr0dc8meaabaqaciaacaGaaeqabaqabeGadaaakeaadaGcaaqaaiabiAda2aWcbeaaaaa@2DC1@, which reflects the relationship among *Y*_1_, *Y*_2 _and *Y*_3_.

According to the Poisson model, *E*(*Z*_*it*_) = *E*(***Y***_*i*_)**e**_*t *_= (*λ*_*i*_(1)*θ*_*i*_,..., *λ*_*i*_(*T*)*θ*_*i*_)**e**_*t*_, *Var*(*Z*_*i*t_) = (*λ*_*i*_(1)*θ*_*i*_,..., *λ*_*i*_(*T*)*θ*_*i*_)et2
 MathType@MTEF@5@5@+=feaafiart1ev1aaatCvAUfKttLearuWrP9MDH5MBPbIqV92AaeXatLxBI9gBaebbnrfifHhDYfgasaacH8akY=wiFfYdH8Gipec8Eeeu0xXdbba9frFj0=OqFfea0dXdd9vqai=hGuQ8kuc9pgc9s8qqaq=dirpe0xb9q8qiLsFr0=vr0=vr0dc8meaabaqaciaacaGaaeqabaqabeGadaaakeaaieqacqWFLbqzdaqhaaWcbaGaemiDaqhabaGaeGOmaidaaaaa@3095@ and *Cov*(*Z*_*it*_, *Z*_*ik*_) = 0 when *t *≠ *k*. Then for a cluster consisting of tags 1, 2,..., *m*, we can measure the cluster dispersion by:

Strans_N=∑i∑t=1,..,T(Zit−E(Zit))2/Var(Zit)=∑i∑t=2,..,T(Zit−(λ^(1)θ^i,...,λ^(T)θ^i)et)2/(λ^(1)θ^i,...,λ^(T)θ^i)et2.     (6)
 MathType@MTEF@5@5@+=feaafiart1ev1aaatCvAUfKttLearuWrP9MDH5MBPbIqV92AaeXatLxBI9gBaebbnrfifHhDYfgasaacH8akY=wiFfYdH8Gipec8Eeeu0xXdbba9frFj0=OqFfea0dXdd9vqai=hGuQ8kuc9pgc9s8qqaq=dirpe0xb9q8qiLsFr0=vr0=vr0dc8meaabaqaciaacaGaaeqabaqabeGadaaakeaafaqadeGabaaabaGaem4uam1aaSbaaSqaaiabdsha0jabdkhaYjabdggaHjabd6gaUjabdohaZjabc+faFjabd6eaobqabaGccqGH9aqpdaWcgaqaamaaqafabaWaaabuaeaadaqadaqaaiabdQfaAnaaBaaaleaacqWGPbqAcqWG0baDaeqaaOGaeyOeI0Iaemyrau0aaeWaaeaacqWGAbGwdaWgaaWcbaGaemyAaKMaemiDaqhabeaaaOGaayjkaiaawMcaaaGaayjkaiaawMcaamaaCaaaleqabaGaeGOmaidaaaqaaiabdsha0jabg2da9iabigdaXiabcYcaSiabc6caUiabc6caUiabcYcaSiabdsfaubqab0GaeyyeIuoaaSqaaiabdMgaPbqab0GaeyyeIuoaaOqaaiabdAfawjabdggaHjabdkhaYnaabmaabaGaemOwaO1aaSbaaSqaaiabdMgaPjabdsha0bqabaaakiaawIcacaGLPaaaaaaabaWaaSGbaeaacqGH9aqpdaaeqbqaamaaqafabaWaaeWaaeaacqWGAbGwdaWgaaWcbaGaemyAaKMaemiDaqhabeaakiabgkHiTmaabmaabaWaaecaaeaaiiGacqWF7oaBaiaawkWaamaabmaabaGaeGymaedacaGLOaGaayzkaaWaaecaaeaacqWF4oqCaiaawkWaamaaBaaaleaacqWGPbqAaeqaaOGaeiilaWIaeiOla4IaeiOla4IaeiOla4IaeiilaWYaaecaaeaacqWF7oaBaiaawkWaamaabmaabaGaemivaqfacaGLOaGaayzkaaWaaecaaeaacqWF4oqCaiaawkWaamaaBaaaleaacqWGPbqAaeqaaaGccaGLOaGaayzkaaacbeGae4xzau2aaSbaaSqaaiabdsha0bqabaaakiaawIcacaGLPaaadaahaaWcbeqaaiabikdaYaaaaeaacqWG0baDcqGH9aqpcqaIYaGmcqGGSaalcqGGUaGlcqGGUaGlcqGGSaalcqWGubavaeqaniabggHiLdaaleaacqWGPbqAaeqaniabggHiLdaakeaadaqadaqaamaaHaaabaGae83UdWgacaGLcmaadaqadaqaaiabigdaXaGaayjkaiaawMcaamaaHaaabaGae8hUdehacaGLcmaadaWgaaWcbaGaemyAaKgabeaakiabcYcaSiabc6caUiabc6caUiabc6caUiabcYcaSmaaHaaabaGae83UdWgacaGLcmaadaqadaqaaiabdsfaubGaayjkaiaawMcaamaaHaaabaGae8hUdehacaGLcmaadaWgaaWcbaGaemyAaKgabeaaaOGaayjkaiaawMcaaiab+vgaLnaaDaaaleaacqWG0baDaeaacqaIYaGmaaaaaOGaeiOla4caaiaaxMaacaWLjaWaaeWaaeaacqaI2aGnaiaawIcacaGLPaaaaaa@B13C@

We should note the connection between this measure and the *S*_*trans *_in formula (5). As we discussed above, the component associated with **e**_2 _is (*Y*_1_-*Y*_2_)/2
 MathType@MTEF@5@5@+=feaafiart1ev1aaatCvAUfKttLearuWrP9MDH5MBPbIqV92AaeXatLxBI9gBaebbnrfifHhDYfgasaacH8akY=wiFfYdH8Gipec8Eeeu0xXdbba9frFj0=OqFfea0dXdd9vqai=hGuQ8kuc9pgc9s8qqaq=dirpe0xb9q8qiLsFr0=vr0=vr0dc8meaabaqaciaacaGaaeqabaqabeGadaaakeaadaGcaaqaaiabikdaYaWcbeaaaaa@2DB9@. Thus the new space associated with *S*_*trans *_is equivalent to the space determined by **e**_2 _and all its row-switching transformations. We can also define a measure similarly through **e**_3 _or other eigenvectors. *S*_*trans *_seems to have the potential of losing the information carried by **e**_3 _and other eigenvectors. However, applications of *TransChisq *to a variety of datasets suggested that this potential information loss is minor and can be ignored in most cases in practice. In fact, the row-switching transformations of **e**_2 _make up most of the information included in **e**_3 _and other eigenvectors.

A potential shortcoming of *S*_*trans_N *_comes from the fact that it is defined based on only one set of eigenvectors. The orthonormal eigenspace of a covariance matrix is not unique (e.g., the row switching operation can result in a different set of eigenvectors) and different eigenspaces may result in different values of *S*_*trans_N *_. Although one can consider all possible eigenspaces to overcome the limitation of *S*_*trans_N*_, it is not computationally feasible.

Applying *S*_*trans_N *_to several different datasets, we observed that i) using the eigenvectors **e**_1_,**e**_2_,...,**e**_*T *_in Additional file 4, *S*_*trans_N *_performs very similarly to *S*_*trans *_and ii) when a different set of eigenvectors used, the clustering results can be different, though the difference is not obvious. These results are not presented in this paper.

### Proposed distance measures (III): PCAChisq

For comparison purposes, we applied PCA to transform the data [19]. PCA is useful to simplify the analysis of a high dimensional dataset. Recently, PCA has been explored as a method for clustering gene expression data [28-33]. But a blind application of PCA in clustering analysis is dangerous in that PCA chooses principal component axes based on the empirical covariance matrix rather than the class information, and thus it does not necessarily give good clustering results [29,34,35].

In some theoretical [35] and empirical [29] studies, there have been observations that the first few principal components (PCs) in PCA are not always helpful to extract meaningful signals from data. Thus, we considered all PCs in this study. By substituting the **e**_1 _**e**_2_...**e**_*T *_in measure (6) by the eigenvectors from the sample covariance matrix, we defined a new measure and implemented it in the *PCAChisq*. The Results section gives examples showing the positive and negative effects of the PCA transformation. In general, *PCAChisq *is difficult to use. Firstly, it is unclear what types of variances the principal components are capturing (if it is the within-cluster variance, the principal components would lead to wrong clustering results). Next, it is unclear how many principal components should be used. The optimal number of PCs is unavailable before we compare the results to the ground truth. To be brief, *PCAChisq *is only efficient when the principal components happen to match the key features that determine a cluster.

### Clustering analysis of microarray data

We explored the potential application of the proposed measures to a clustering analysis of microarray data. We proposed the following restricted normal model for this purpose. The parameter notations in the Poisson model were adopted. Given a microarray dataset of expressions of *n *genes in *T *experiments, the expression of gene *i *in experiment *t*, *X*_*i*_(*t*), is assumed to be normally distributed with mean *μ*_*i*_(*t*) = *λ*_*i*_(*t*)*θ*_*i *_and variance σi2
 MathType@MTEF@5@5@+=feaafiart1ev1aaatCvAUfKttLearuWrP9MDH5MBPbIqV92AaeXatLxBI9gBaebbnrfifHhDYfgasaacH8akY=wiFfYdH8Gipec8Eeeu0xXdbba9frFj0=OqFfea0dXdd9vqai=hGuQ8kuc9pgc9s8qqaq=dirpe0xb9q8qiLsFr0=vr0=vr0dc8meaabaqaciaacaGaaeqabaqabeGadaaakeaaiiGacqWFdpWCdaqhaaWcbaGaemyAaKgabaGaeGOmaidaaaaa@30F0@(*t*) = *kλ*_*i*_(*t*)*θ*_*i*_, where *k *is an unknown constant. The derivation of the maximum likelihood estimates (MLEs) of *λ*_*i*_(*t*) and *θ*_*i *_under the normal model is rather involved. So we borrowed the estimators in formula (2). It can be shown that θ^
 MathType@MTEF@5@5@+=feaafiart1ev1aaatCvAUfKttLearuWrP9MDH5MBPbIqV92AaeXatLxBI9gBaebbnrfifHhDYfgasaacH8akY=wiFfYdH8Gipec8Eeeu0xXdbba9frFj0=OqFfea0dXdd9vqai=hGuQ8kuc9pgc9s8qqaq=dirpe0xb9q8qiLsFr0=vr0=vr0dc8meaabaqaciaacaGaaeqabaqabeGadaaakeaadaqiaaqaaGGaciab=H7aXbGaayPadaaaaa@2F2B@_*i *_in formula (2) is unbiased and λ^
 MathType@MTEF@5@5@+=feaafiart1ev1aaatCvAUfKttLearuWrP9MDH5MBPbIqV92AaeXatLxBI9gBaebbnrfifHhDYfgasaacH8akY=wiFfYdH8Gipec8Eeeu0xXdbba9frFj0=OqFfea0dXdd9vqai=hGuQ8kuc9pgc9s8qqaq=dirpe0xb9q8qiLsFr0=vr0=vr0dc8meaabaqaciaacaGaaeqabaqabeGadaaakeaadaqiaaqaaGGaciab=T7aSbGaayPadaaaaa@2F29@_*t *_in formula (2) is consistent under the restricted normal model [see Additional file 5]. With θ^
 MathType@MTEF@5@5@+=feaafiart1ev1aaatCvAUfKttLearuWrP9MDH5MBPbIqV92AaeXatLxBI9gBaebbnrfifHhDYfgasaacH8akY=wiFfYdH8Gipec8Eeeu0xXdbba9frFj0=OqFfea0dXdd9vqai=hGuQ8kuc9pgc9s8qqaq=dirpe0xb9q8qiLsFr0=vr0=vr0dc8meaabaqaciaacaGaaeqabaqabeGadaaakeaadaqiaaqaaGGaciab=H7aXbGaayPadaaaaa@2F2B@_*i *_and λ^
 MathType@MTEF@5@5@+=feaafiart1ev1aaatCvAUfKttLearuWrP9MDH5MBPbIqV92AaeXatLxBI9gBaebbnrfifHhDYfgasaacH8akY=wiFfYdH8Gipec8Eeeu0xXdbba9frFj0=OqFfea0dXdd9vqai=hGuQ8kuc9pgc9s8qqaq=dirpe0xb9q8qiLsFr0=vr0=vr0dc8meaabaqaciaacaGaaeqabaqabeGadaaakeaadaqiaaqaaGGaciab=T7aSbGaayPadaaaaa@2F29@_*t *_available under the normal model, *TransChisq*, *PCAChisq *and *PoissonC *can be applied.

For both oligonucleotide and cDNA microarray data, it is widely observed that there is strong dependence of the variance on the mean: variance increases with mean [36,37]. So it is reasonable to expect that our restricted normal model is applicable to many microarray datasets. One example of this application on the yeast sporulation dataset has been presented to demonstrate the power of *TransChisq *in analyzing microarray data (see the Results section). We should also note that *TransChisq *would deliver less promising results if the assumption on the relationship between the variance and the mean is seriously violated.

## Authors' contributions

KK participated in the design of the study, performed the analysis and drafted the Results section of the manuscript. SZ, KJ and LJF provided the Maize root microarray data, which helped in motivating this research. SZ, KJ and LJF were responsible for the biological explanations on the results related to maize data. LC provided the developing mouse retina SAGE data and was responsible to the biological explanations on the clustering results related to SAGE data. IBL helped in formulating PCA related studies. HH conceived of this study, proposed the method, coordinated the collaborations and wrote the paper. All authors read and approved the final manuscript.

## Supplementary Material

Additional File 1 **One set of orthonormal eigenvectors**. This PDF file contains one set of orthonormal eigenvectors referred in the Method section. Click here for file

Additional File 2 **Proof of the properties of the estimators under the restricted normal model**. This PDF file shows that the θ^
 MathType@MTEF@5@5@+=feaafiart1ev1aaatCvAUfKttLearuWrP9MDH5MBPbIqV92AaeXatLxBI9gBaebbnrfifHhDYfgasaacH8akY=wiFfYdH8Gipec8Eeeu0xXdbba9frFj0=OqFfea0dXdd9vqai=hGuQ8kuc9pgc9s8qqaq=dirpe0xb9q8qiLsFr0=vr0=vr0dc8meaabaqaciaacaGaaeqabaqabeGadaaakeaadaqiaaqaaGGaciab=H7aXbGaayPadaaaaa@2F2B@_*i *_in formula (2) is an unbiased estimator of *θ*_*i *_and λ^
 MathType@MTEF@5@5@+=feaafiart1ev1aaatCvAUfKttLearuWrP9MDH5MBPbIqV92AaeXatLxBI9gBaebbnrfifHhDYfgasaacH8akY=wiFfYdH8Gipec8Eeeu0xXdbba9frFj0=OqFfea0dXdd9vqai=hGuQ8kuc9pgc9s8qqaq=dirpe0xb9q8qiLsFr0=vr0=vr0dc8meaabaqaciaacaGaaeqabaqabeGadaaakeaadaqiaaqaaGGaciab=T7aSbGaayPadaaaaa@2F29@(*t*) in formula (2) is a consistent estimator of *λ*(*t*) under the proposed restricted normal model. Click here for file

Additional File 3 **The performance of new measures in a hierarchical clustering algorithm**. This PDF file presents the application results of the hierarchical clustering algorithms with different measures implemented. Click here for file

Additional File 4 **The effects of the *TransChisq *data transformation in measuring pattern similarity**. This PDF file presents a simple simulation study for the effects of the data transformation in *TransChisq *with a comparison to *PoissonC*. Click here for file

Additional File 5 **The guideline on the various parameters in the simulation dataset in **Table 2. This PDF file presents the motivation and guideline for choosing the various parameters in the simulation dataset in Table 2. Click here for file
