# Supplementary material for: Measuring similarities between gene expression profiles through new data transformations
Source: BMC Bioinformatics. 2007 Jan 27;8:29. doi: 10.1186/1471-2105-8-29 (PMC1804284; doi:10.1186/1471-2105-8-29)
Supplement: Additional File 1 — One set of orthonormal eigenvectors. This PDF file contains one set of orthonormal eigenvectors referred in the Method section. [file 1471-2105-8-29-S1.pdf]

Additional File 1.

The following summarizes the motivation and guideline for choosing the various parameters in the five dimensional simulation dataset in Table 2:

- In our study, the mouse SAGE dataset is of dimension 10, the yeast Microarray dataset is of dimension 7 and the maize dataset is of dimension 3. So we intended to choose a number close to 7. We simulated two datasets. One has dimension 5 and the other one has dimension 7. The results and story from the two datasets are very similar. As the advantages and disadvantages of different methods can be seen more clearly from a lower dimensional dataset, so we chose to present the results on the 5 dimensional simulation dataset in this paper.
- The number of groups, 6, was chosen because the two experimental datasets (mouse SAGE data and yeast Microarray data) we considered consist of 5 and 7 functional gene groups respectively. So we used the average number.
- The number of members in each group was determined by the following consideration: to show the power of the method, we included a variety of clusters of very different sizes in the data (e.g., group A in the simulation dataset is a small cluster with 3 members, and group F is a large cluster with 15 members). Using "3" as the size of the small cluster and "15" as the size of the large cluster are somewhat arbitrary.
- The patterns of each group were determined based on the patterns observed from the experimental datasets. The means of the Poisson distributions were determined by two rules: i) Include profiles with different magnitudes within a cluster, e.g., in group D, the magnitude of vectors d1-d7 is much smaller than that of vectors d8 and d9, though they share the same shape profile; ii) The shape profiles are similar but not exactly the same within a cluster, e.g., in group F, the shape parameter of vectors f1, f2 and f3 is $\boldsymbol{\lambda} = (0.07, 0.71, 0.07, 0.07, 0.07)$ and the shape parameter of vectors f7, f8 and f9 is $\boldsymbol{\lambda} = (0.04, 0.83, 0.04, 0.04, 0.04)$.
