# Supplementary material for: Measuring similarities between gene expression profiles through new data transformations
Source: BMC Bioinformatics. 2007 Jan 27;8:29. doi: 10.1186/1471-2105-8-29 (PMC1804284; doi:10.1186/1471-2105-8-29)
Supplement: Additional File 2 — Proof of the properties of the estimators under the restricted normal model. This PDF file shows that the θ^ MathType@MTEF@5@5@+=feaafiart1ev1aaatCvAUfKttLearuWrP9MDH5MBPbIqV92AaeXatLxBI9gBaebbnrfifHhDYfgasaacH8akY=wiFfYdH8Gipec8Eeeu0xXdbba9frFj0=OqFfea0dXdd9vqai=hGuQ8kuc9pgc9s8qqaq=dirpe0xb9q8qiLsFr0=vr0=vr0dc8meaabaqaciaacaGaaeqabaqabeGadaaakeaadaqiaaqaaGGaciab=H7aXbGaayPadaaaaa@2F2B@i in formula (2) is an unbiased estimator of θi and λ^ MathType@MTEF@5@5@+=feaafiart1ev1aaatCvAUfKttLearuWrP9MDH5MBPbIqV92AaeXatLxBI9gBaebbnrfifHhDYfgasaacH8akY=wiFfYdH8Gipec8Eeeu0xXdbba9frFj0=OqFfea0dXdd9vqai=hGuQ8kuc9pgc9s8qqaq=dirpe0xb9q8qiLsFr0=vr0=vr0dc8meaabaqaciaacaGaaeqabaqabeGadaaakeaadaqiaaqaaGGaciab=T7aSbGaayPadaaaaa@2F29@(t) in formula (2) is a consistent estimator of λ(t) under the proposed restricted normal model. [file 1471-2105-8-29-S2.pdf]

Additional File 2.

To further evaluate our new measures, we implemented them and other commonly used ones in the agglomerative hierarchical clustering procedure. For ease of notation, we call the hierarchical algorithms as *TransChisq*, *PCAChisq*, *PoissonC*, *PearsonC* and *Eucli* (based on what measures used) as well. When we calculate the pair's distance by *TransChisq*, *PCAChisq*, and *PoissonC*, we assume that two genes form one cluster. Once clustering commences, we need work with items that are true items (e.g. a single gene) and items that are pseudo-items that contain a number of true items. We applied different ways to compute distances when we are dealing with pseudo-items: ward method, complete linkage, single linkage, and average linkage. Table A1 and A2 show the application results of the varied hierarchical clustering algorithms to the two experimental datasets. For the Microarray yeast sporulation data, each hierarchical tree was cut at K=7 to obtain 7 clusters. For the SAGE data, the tree was cut at K=5. Adjusted Rand Index was used to evaluate the clustering results. We can see that, in general, single linkage and average linkage generate worse clustering results compared to Ward's method and complete linkage. For the yeast data, the combination of *TransChisq* and the Ward's method give the best result. For the SAGE data, *TransChisq* and *PoissonC* combined with the Ward's method perform similarly and outperform other combinations. This study shows that our new measure is still efficient when it is implemented in the hierarchical clustering algorithm.

Table A1. Hierarchical clustering results on the 39 yeast sporulation genes.

	Adjusted Rand Index			
	Ward	Complete	Single	Average
TransChisq	**0.830**	0.725	0.110	0.278
PCAChisq	0.372	0.725	0.110	0.372
PoissonC	0.495	0.496	0.110	0.628
PearsonC	0.552	0.464	0.244	0.483
Eucli	0.600	0.420	0.112	0.396
Eucli on rescaled data	0.539	0.446	0.119	0.367

Table A2. Hierarchical clustering results on the 153 SAGE tags.

	Adjusted Rand Index			
	Ward	Complete	Single	Average
TransChisq	**0.778**	0.544	0.000	0.358
PCAChisq	0.542	0.505	0.000	0.406
PoissonC	**0.788**	0.432	0.000	0.511
PearsonC	0.527	0.659	0.004	0.565
Eucli	0.004	0.000	0.000	0.000
Eucli on rescaled data	0.627	0.417	0.348	0.373
