# Supplementary material for: Measuring similarities between gene expression profiles through new data transformations
Source: BMC Bioinformatics. 2007 Jan 27;8:29. doi: 10.1186/1471-2105-8-29 (PMC1804284; doi:10.1186/1471-2105-8-29)
Supplement: Additional File 3 — The performance of new measures in a hierarchical clustering algorithm. This PDF file presents the application results of the hierarchical clustering algorithms with different measures implemented. [file 1471-2105-8-29-S3.pdf]

Additional File 3.

For an object with expression profile $\boldsymbol{Y} = (Y_1,\ldots,Y_T)$, we denote its expected profile in a certain cluster by $E(\boldsymbol{Y}) = (E(Y_1),\ldots,E(Y_T))$. We denote the expression profile and its expectation in a transformed space by $\boldsymbol{Z} = \boldsymbol{Y}A = (Z_1,\ldots,Z_n)$ and $E(\boldsymbol{Z}) = E(\boldsymbol{Y}A) = E(\boldsymbol{Y})$ respectively, where A is the transformation matrix. *PoissonC* measures the departure of $\boldsymbol{Y}$ from $E(\boldsymbol{Y})$, while *TransChisq* and *PCAChisq* measure the departure of $\boldsymbol{Z}$ from $E(\boldsymbol{Z})$. The characteristic of the transformation matrix determines the property of the measure. The transformation matrix in *TransChisq* is made up by all the row-switching vectors of $\mathbf{e}_2 = [1/\sqrt{2}, -1/\sqrt{2}, 0, \ldots, 0]^T$ (see page 12 and 13 of the paper), so *TransChisq* puts more emphasis on the relationship between components in the original space. The transformation matrix in *PCAChisq* is made up by the eigenvectors of the sample covariance matrix (representing the principal component space). So *PCAChisq* focuses more on the features captured by the principal components.

The tradeoff between magnitude and shape in *TransChisq* can be learnt from the simulation study shown in Table A3. Vectors V_1, V_2 and V_3 are randomly generated by four Poisson distributions with means $\theta_1\boldsymbol{\lambda}$ (θ_1=900 and $\boldsymbol{\lambda}$ = (1/6, 1/3, 1/6, 1/3)). V_4, V_5 and V_6 are randomly generated by Poisson distributions with the means specified by the same $\boldsymbol{\lambda}$ but a different magnitude parameter θ_2=45. The distances of the simulated vectors to four different clusters are calculated by both S_{trans} (the measure (5) used in *TransChisq*) and D (the measure (4) used in *PoissonC*). The shape parameters of the four clusters together with the calculated distances are shown in Table A3. Three interesting observations can be found from Table A3.

- Comparing Table A3(a) with A3(b), the key difference between *TransChisq* and *PoissonC* is in the distances of the vectors to Cluster I and Cluster II. *PoissonC* determines that the six vectors have similar distances to Cluster I and II whereas *TransChisq* favors Cluster II. The result from *TransChisq* makes more sense if there is an emphasis on shape in determining the relationship. Cluster II has a similar pattern to the six vectors' as they all show relatively high values on the second and fourth components. Cluster I has a totally opposite pattern to that of the six vectors.
- From Table A3(a), we see that the distances of vectors V_1, V_2 and V_3 to Cluster I, II and III are at a much larger scale than the distances of V_4, V_5 and V_6 to the clusters I-III. Note that the magnitude of V_1, V_2 and V_3 is much larger (θ_1=900) than that of V_4, V_5 and V_6 (θ_2=45) and the patterns of Clusters I, II and III are all different from the six vectors'. It means that the penalty from assigning the objects with larger magnitude to a wrong cluster is more than that from the objects with a smaller magnitude. In other words, *TarnsChisq* is less tolerant to the **shape change** for objects with larger values. This is reasonable in that the estimation of shape parameter for objects with smaller magnitude is less precise than the estimation from objects with larger expression values. Thus, the shape change of objects with smaller magnitude should be taken less seriously. As a result, we can see that the distances of vectors V_1, V_2 and V_3 to Cluster III (with a pattern very close to the true pattern of V_1, V_2 and V_3) is

similar to the distances of V_4, V_5 and V_6 to Cluster I (with an opposite pattern to the true pattern of V_4, V_5 and V_6).

- From the last row of Figure A3(a), we can see that the distances of vectors V_1, V_2 and V_3 to Cluster IV are similar to the distances of vectors V_4, V_5 and V_6 to Cluster IV.. The magnitude difference among the vectors seems not matter. This happens because the pattern of Cluster IV is the same as the true pattern of the six vectors. The observed distances are actually due to the distribution variance.

Table A3 (a). ***TransChisq*** **distances to clusters with different patterns**

TransChisq	θ_1=900<br>λ^*=(1/6,1/3,1/6,1/3)			θ_2=45<br>λ^*=(1/6,1/3,1/6,1/3)		
Cluster shape profile	V1	V2	V3	V4	V5	V6
**Cluster I**: λ1=(1/3,1/6,1/3,1/6)	842.58	769.49	824.21	58.735	41.353	48.856
**Cluster II**: λ2=(1/18,4/9,1/18,4/9)	333.11	363.38	358.35	19.302	27.373	40.626
**Cluster III**: λ3=(1/8,3/8,1/8,3/8)	45.58	57.958	56.578	3.9967	6.2712	16.482
**Cluster IV**: λ4=(1/6,1/3,1/6,1/3)	5.8226	4.9145	11.631	3.3529	1.7941	10.289

Table A3 (b). ***PoissonC*** **distances to clusters with different patterns**

PoissonC	θ_1=900<br>λ^*=(1/6,1/3,1/6,1/3)			θ_2=45<br>λ^*=(1/6,1/3,1/6,1/3)		
Cluster shape profile	V1	V2	V3	V4	V5	V6
**Cluster I**: λ1=(1/3,1/6,1/3,1/6)	474.2	433.07	462.55	32.824	23.118	27.964
**Cluster II**: λ2=(1/18,4/9,1/18,4/9)	417.23	455.98	442.31	21.794	33.044	43.918
**Cluster III**: λ3=(1/8,3/8,1/8,3/8)	29.1	37.468	37.718	2.5948	4.1634	8.6606
**Cluster IV**: λ4=(1/6,1/3,1/6,1/3)	2.6067	2.1549	6.9696	1.9412	1.0588	4.5636
