# Supplementary material for: Measuring similarities between gene expression profiles through new data transformations
Source: BMC Bioinformatics. 2007 Jan 27;8:29. doi: 10.1186/1471-2105-8-29 (PMC1804284; doi:10.1186/1471-2105-8-29)
Supplement: Additional File 4 — The effects of the TransChisq data transformation in measuring pattern similarity. This PDF file presents a simple simulation study for the effects of the data transformation in TransChisq with a comparison to PoissonC. [file 1471-2105-8-29-S4.pdf]

Additional File 4.

One set of T column eigenvector of the covariance matrix in (6) is given by

$$\begin{bmatrix}\mathbf{e}_1 & \cdots & \mathbf{e}_T\end{bmatrix} = \left[\begin{array}{c|ccc} 1 & -1 & \cdots & -1 \\ \hline 1 & & & \\ \vdots & & \mathbf{I}_{(T-1)\times(T-1)} & \\ 1 & & & \end{array}\right], \tag{S1}$$

where $\mathbf{I}_{(T-1)\times(T-1)}$ is an (T-1)-dimensional identity matrix. Orthonormal eigenvectors can be further obtained by applying the Gramm-Schmidt procedure which orthogonalize each eigenvector ($\mathbf{e}_i$) with respect to all the other eigenvectors to give $\mathbf{e}_{i\perp}$:

$$\mathbf{e}_{i\perp} = \frac{\mathbf{e}_i - \sum_{j=1}^{i-1}\left(\mathbf{e}_i \circ \mathbf{e}_{j\perp}\right)\mathbf{e}_{j\perp}}{\left|\mathbf{e}_i - \sum_{j=1}^{i-1}\left(\mathbf{e}_i \circ \mathbf{e}_{j\perp}\right)\mathbf{e}_{j\perp}\right|} \tag{S2}$$

Finally, we can obtain one set of orthonormal eigenvectors of (S1) as follows:

$$\begin{bmatrix}\mathbf{e}_{1\perp} & \mathbf{e}_{2\perp} & \mathbf{e}_{3\perp} & \cdots & \mathbf{e}_{T\perp}\end{bmatrix} = \begin{bmatrix} \frac{1}{\sqrt{T}} & \frac{1}{\sqrt{2}} & \frac{1}{\sqrt{6}} & \cdots & \frac{1}{\sqrt{T(T-1)}} \\ \frac{1}{\sqrt{T}} & -\frac{1}{\sqrt{2}} & \frac{1}{\sqrt{6}} & \cdots & \frac{1}{\sqrt{T(T-1)}} \\ \frac{1}{\sqrt{T}} & 0 & -\frac{2}{\sqrt{6}} & \cdots & \frac{1}{\sqrt{T(T-1)}} \\ \frac{1}{\sqrt{T}} & 0 & 0 & \cdots & \frac{1}{\sqrt{T(T-1)}} \\ \vdots & \vdots & \vdots & \ddots & \vdots \\ \frac{1}{\sqrt{T}} & 0 & 0 & \cdots & -\frac{T-1}{\sqrt{T(T-1)}} \end{bmatrix} \tag{S3}$$

For ease of notation, we used $\mathbf{e}_i$ instead of $\mathbf{e}_{i\perp}$ in the main text to denote the orthonormal eigenvectors. By randomly permuting the rows (performing row-switching transformations), we can obtain alternative orthonormal eigenspaces of the covariance matrix in (6).
