# Supplementary material for: Measuring similarities between gene expression profiles through new data transformations
Source: BMC Bioinformatics. 2007 Jan 27;8:29. doi: 10.1186/1471-2105-8-29 (PMC1804284; doi:10.1186/1471-2105-8-29)
Supplement: Additional File 5 — The guideline on the various parameters in the simulation dataset in Table 2. This PDF file presents the motivation and guideline for choosing the various parameters in the simulation dataset in Table 2. [file 1471-2105-8-29-S5.pdf]

Additional File 5.

Let the expression of gene i at experiment t, $X_i(t)$, follow a Normal distribution with mean $\lambda(t)\theta_i$ and variance $k\lambda(t)\theta_i$, where k is a constant. We want to show that the $\widehat{\theta}_i$ in (2) is an unbiased estimator of θ_i and $\hat{\lambda}(t)$ in (2) is a consistent estimator of $\lambda(t)$ under this normal model. By (2), $\widehat{\theta}_i$ and $\hat{\lambda}(t)$ can be computed by

$$\widehat{\theta}_i = \sum_{t=1}^{T} X_i(t),\ \hat{\lambda}(t) = \frac{\sum_{i=1}^{n} X_i(t)}{\sum_{i=1}^{n}\sum_{t=1}^{T} X_i(t)}. \tag{S4}$$

Statement 1. $\widehat{\theta}_i$ is unbiased.

Proof: $E\left(\widehat{\theta}_i\right) = \sum_{t=1}^{T} E\left(X_i(t)\right) = \sum_{t=1}^{T}\left(\lambda(t)\theta_i\right) = \theta_i \sum_{t=1}^{T}\lambda(t) = \theta_i$. So $\widehat{\theta}_i$ is an unbiased estimator of θ_i. ▮

Statement 2. $\hat{\lambda}(t)$ is a consistent estimator of $\lambda(t)$.

Proof: For $\hat{\lambda}(t)$ to be a consistent estimator of $\lambda(t)$, it is sufficient to show that $\hat{\lambda}(t)-\lambda(t)$ converges to 0 in probability. By (S4), we have

$$\hat{\lambda}(t)-\lambda(t) = \frac{\sum_{i=1}^{n} X_i(t) - \lambda(t)\sum_{i=1}^{n}\sum_{j=1}^{T} X_i(j)}{\sum_{i=1}^{n}\sum_{j=1}^{T} X_i(j)} = \frac{\frac{1}{n}\left[\sum_{i=1}^{n} X_i(t) - \lambda(t)\sum_{i=1}^{n}\sum_{j=1}^{T} X_i(j)\right]}{\frac{1}{n}\sum_{i=1}^{n}\sum_{j=1}^{T} X_i(j)}. \tag{S5}$$

We first consider the numerator (M_n) of (S5).

$$\begin{aligned} E(M_n) &= \frac{1}{n}\sum_{i=1}^{n}\left[\lambda(t)\theta_i - \lambda(t)\left(\sum_{j=1}^{T}\lambda(j)\theta_i\right)\right] \\ &= \frac{1}{n}\sum_{i=1}^{n}\left[\lambda(t)\theta_i - \lambda(t)\theta_i\right] \\ &= 0 \end{aligned},$$

and

$$
\begin{aligned}
Var(M_n) &= \frac{1}{n^2}\sum_{i=1}^{n}\left[\left(1-\lambda(t)\right)^2 k\lambda(t)\theta_i + \lambda(t)^2\left(\sum_{j\neq t}^{T} k\lambda(j)\theta_i\right)\right] \\
&= \frac{1}{n^2}\sum_{i=1}^{n}\left[\left(1-\lambda(t)\right)^2 k\lambda(t)\theta_i + k\lambda(t)^2\left(1-\lambda(t)\right)\theta_i\right] \\
&= \frac{1}{n^2}\sum_{i=1}^{n}\left[k\left(1-\lambda(t)\right)\lambda(t)\theta_i\right] \\
&= \frac{k\left(1-\lambda(t)\right)\lambda(t)\theta_i}{n}
\end{aligned}.
$$

So the numerator M_n converges to 0 in probability as n goes to infinity. Now we consider the denominator (D_n) in (S5). It is reasonable to assume that θ_i's are uniformly bounded. That is that there exists a positive real value A and B, such that $A \le |\theta_i| \le B$ for any i. Then we have

$$0 < A \le E(D_n) = \frac{\sum_{i=1}^{n}\theta_i}{n} \le B,\ \ Var(D_n) = \frac{\sum_{i=1}^{n}k\theta_i}{n^2} \le \frac{kB}{n} \text{ and } \lim_{n\to\infty} Var(D_n) = 0.$$

Consequently, for any $\varepsilon > 0$, we have

$$
\begin{aligned}
\lim_{n\to\infty} P\left(\left|\tfrac{M_n}{D_n}\right| > \varepsilon\right) &= \lim_{n\to\infty}\left(P\left(\left|\tfrac{M_n}{D_n}\right| > \varepsilon, | D_n - E(D_n) | > \tfrac{A}{2}\right) + P\left(\left|\tfrac{M_n}{D_n}\right| > \varepsilon, | D_n - E(D_n) | \le \tfrac{A}{2}\right)\right) \\
&\le \lim_{n\to\infty}\left(P\left(| D_n - E(D_n) | > \tfrac{A}{2}\right) + P\left(\left|\tfrac{M_n}{A/2}\right| > \varepsilon, | D_n - E(D_n) | \le \tfrac{A}{2}\right)\right) \\
&\le \lim_{n\to\infty}\left(\tfrac{Var(D_n)}{(A/2)^2} + P\left(| M_n | > \tfrac{A\varepsilon}{2}\right)\right) \\
&= \lim_{n\to\infty}\tfrac{Var(D_n)}{(A/2)^2} + \lim_{n\to\infty} P\left(| M_n | > \tfrac{A\varepsilon}{2}\right) \\
&= 0
\end{aligned},
$$

So $\hat{\lambda}(t) - \lambda(t) = \frac{M_n}{D_n}$ converges to 0 in probability as n goes to infinity, and then $\hat{\lambda}(t)$ is a consistent estimator of $\lambda(t)$. ∎
